# Structural Manipulation of the Conjugated Phenyl Moiety in 3-Phenylbenzofulvene Monomers: Effects on Spontaneous Polymerization

**DOI:** 10.3390/polym10070752

**Published:** 2018-07-07

**Authors:** Marco Paolino, Giorgio Grisci, Annalisa Reale, Vincenzo Razzano, Germano Giuliani, Alessandro Donati, Raniero Mendichi, Daniele Piovani, Antonella C. Boccia, Alessandro Grillo, Gianluca Giorgi, Andrea Cappelli

**Affiliations:** 1 Dipartimento di Biotecnologie, Chimica e Farmacia (Dipartimento di Eccellenza 2018-2022), Università degli Studi di Siena, Via Aldo Moro 2, 53100 Siena, Italy; giorgio.grisci@outlook.it (Gio.G.); reale5@student.unisi.it (A.R.); v.razzano88@gmail.com (V.R.); giuliani5@unisi.it (Ge.G.); alessandro.donati@unisi.it (A.D.); grillo.alessandro@ymail.com (A.G.); gianluca.giorgi@unisi.it (Gia.G.); 2 Istituto per lo Studio delle Macromolecole (CNR), Via A. Corti 12, 20133 Milano, Italy; raniero.mendichi@ismac.cnr.it (R.M.); piovani@ismac.cnr.it (D.P.); antonella.boccia@ismac.cnr.it (A.C.B.)

**Keywords:** spontaneous polymerization, affinity polymerization, π-conjugated polymer, through-space conjugated polymer, polybenzofulvene

## Abstract

Spontaneous polymerization is an intriguing phenomenon in which pure monomers begin their polymerization without initiators or catalysts. Previously, 3-phenylbenzofulvene monomers were found to polymerize spontaneously after solvent removal. Here, eight new 3-substituted benzofulvene monomers **1a–h** were synthesized in order to investigate the effects of differently substituted aromatic rings in position 3 of the benzofulvene scaffold on spontaneous polymerization. The newly synthesized monomers maintained the tendency toward spontaneous polymerization. However, monomer **1a**, bearing an ortho-methoxy substituted phenyl, polymerized hardly, thus producing low polymerization yields, inhomogeneous structure, and low molecular weight of the obtained polymeric material. This result suggested the importance of the presence of hydrogen atoms in the 2′-position to achieve productive interactions among the monomers in the recognition step preluding the spontaneous polymerization and among the monomeric units in the polybenzofulvene backbones. Moreover, this study paves the way to modify the pendant rings in position 3 of the indene scaffold to synthesize new polybenzofulvene derivatives variously decorated.

## 1. Introduction

**Figure 1:**
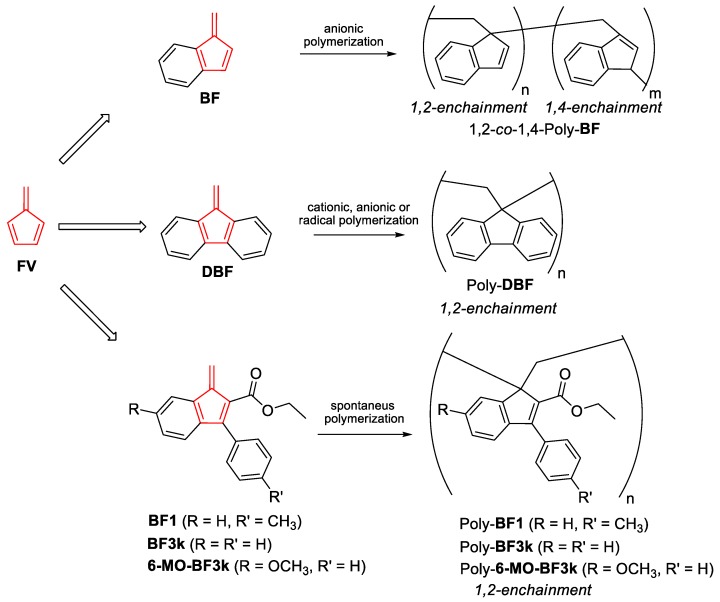
Schematic representation of the most important fulvene monomers and their corresponding polymers.

π-Conjugated polymers are particular materials in which charge and energy are transported through a conjugated polymer backbone [1,2,3]. Since their discovery, π-conjugated polymers have found increasing application interest for the realization of different types of electronic devices [4]. Thanks to their mechanical property, low intrinsic cost, and the ability to manipulate its structure, they are used in the development of organic light-emitting diodes (OLEDs) [5,6], solar and photovoltaic cells [7,8], and sensors and field effect transistors (FETs) [9,10]. A particular subclass of π-conjugated polymers is the “through-space conjugated polymers”, in which π-electron systems (such as aromatic rings) are packaged in the polymer chain but are not directly conjugated through the backbone [11,12]. Among these, poly(*N*-vinylcarbazole) (**PVK**) is the most commonly used because, thanks to the stacked orbitals of its carbazole moieties, it is capable of intrachain charge transport, showing photoconductive, photorefractive, hole-transporting, energy-donating, and carrier-switching properties [13,14]. A similar type of intrachain stacking has been proposed to affect the properties of some fulvene (**FV**) derivative polymers: benzofulvene (**BF**) and dibenzofulvene (**DBF**) polymers (Figure 1) [15].

Fulvene is an isomer of benzene constituted by a five-membered ring and an exocyclic double bond. It represents one among the classical nonalternant π-electron systems that potentially satisfy the Huckel 4N + 2 rule [16]. Due to the polarization of the exocyclic double bond, **FV** shows a strong reactivity towards a large variety of reagents and **FV** derivatives have been polymerized using different techniques [16,17,18]. A particular **FV** derivate is dibenzofulvene (**DBF** or 9-methylene-9*H*-fluorene), in which two benzene rings are symmetrically condensed at two fulvene edges. Nakano and coworkers have extensively studied the behavior of **DBF** as a monomer in anionic, cationic, or radical polymerizations [19,20,21]. These studies clearly demonstrated that **DBF** always adopts a 1,2 vinyl-type polymerization mechanism leading to polymers featuring nearly all-trans backbones and stacked although slightly twisted fluorene units [22]. Furthermore, **DBF** was shown to polymerize spontaneously in the solid state through the reaction between oxygen and the exocyclic double bond [23]. Owing to its high hole mobility, the π-stacked poly-**DBF** has been proposed as a vinyl polymer for electronic applications [24].

On the contrary, benzofulvene (**BF** or 1-methylene-1*H*-indene) shows only one aromatic ring condensed at the fulvene ring. This attractive structure has been recently studied by Ishizone and coworkers as a monomer in living anionic polymerizations [25]. **BF** was demonstrated to polymerize into polymers showing a predictable molecular weight (regulated by the amount of initiator) and a narrow molecular weight distribution (*M*_w_/*M*_n_ = 1.1). However, the structure of the corresponding poly-**BF** derivatives was found to be rather heterogeneous. In fact, **BF** can behave as a 1,3-diene in the anionic polymerization and consequently the obtained polymers were demonstrated to possess, in their backbone, 1,4 and 1,2 sequences equally possible [26,27].

In the last 15 years, our research group has been involved in intensive studies on π-stacked polymers based on variously substituted 3-phenyl-1*H*-indene repeating units [28,29]. Our polybenzofulvene derivatives, which found in poly-**BF1** their prototype, show peculiar features such as high molecular weight, solubility in organic solvents and tunable solubility and assembling in water, propensity to give nanostructured aggregates, and variability in their physicochemical properties on the basis of the substituents on the monomeric units [30,31]. Moreover, the most important and curious property of the 1-methylene-3-phenyl-1*H*-indene derivatives is the spontaneous and thermoreversible polymerization, which occurs by simple removal of the solvent from a solution of the monomer in the apparent absence of initiators or catalysts [32]. In fact, contrary to **BF** and **DBF**, which possess planar structures and are both crystalline solids, the 1-methylene-3-phenyl-1*H*-indene derivatives polymerized spontaneously, leading to the quantitative formation of the corresponding polymers without side reaction or byproducts [32].

The structural manipulation of the monomeric unit of this class of polymers has produced fascinating materials showing unusual properties [2,32]. In particular, interesting new drug delivery systems were developed through the insertion of oligoethylene glycol (OEG) side chain or the conjugation with biocompatible macromolecules [33,34,35,36,37,38,39,40]. Moreover, the structural manipulation of the 3-phenylindene moiety has provided intriguing results in the optoelectronic field, leading to potentially useful polymers in the fabrication of OLED devices [41,42,43,44,45,46,47,48]. However, these results were obtained after intensive structure-property relationship studies were performed on monomer **BF1** and focused on the identification of the effects of different substituents on the polymer properties (i.e., spontaneous polymerization, molecular weight distribution, structure, thermoreversibility, photophysical features, and aggregation). These studies have mainly involved the position 2 (near to the polymerizable exocyclic double bond) and the positions 4, 5, and 6 of indene nucleus. The presence of substituents in positions 4, 5, and 6 did not significantly alter the tendency to spontaneous polymerization of the benzofulvene monomers [2]. In particular, the spontaneous polymerization process is compatible also with the presence of bulky substituents in position 6 of the indene nucleus [49,50]. Nevertheless, a very important role seems to be played by the substituent in position 2. In fact, bulky groups, such as –CON(CH_3_)_3_ or –C(CH_3_)_3_, sterically inhibit spontaneous polymerization. On the other hand, the presence of small substituents (i.e., H, F, Cl, Br, CN) appeared to promote the formation of structurally inhomogeneous polymer chains in which (1,2) and (1,4) sequences seemed to coexist in the main chain. Finally, the electron-withdrawing ethyl ester group was found to be the most interesting substituent because its presence led to the formation of polybenzofulvene derivatives showing preferential (1,2) vinyl structure and high molecular weight [32].

We have previously proposed for the phenyl ring in position 3 of the indene nucleus a pivotal role in the spontaneous polymerization of our benzofulvene monomers. In our hypothesis, in agreement with the crucial importance of specific aromatic interactions in biological [51,52] or synthetic [53,54] macromolecules, the pendant phenyl ring of the 3-phenyl-1*H*-indene monomer was assumed to establish productive specific aromatic (i.e., π-stacking) interactions capable of affecting the aggregation of the monomers leading to the spontaneous polymerization process [29].

In order to better characterize the role of the pendant phenyl substituent in influencing the spontaneous polymerization of benzofulvene monomers and the properties of the corresponding polymers, its structure was manipulated further. Thus, in this paper, we report on the synthesis and the characterization of eight new 3-substituted benzofulvene monomers variously substituted on the pendant phenyl ring and of the corresponding polymeric materials.

## 2. Materials and Methods

### 2.1. Synthesis

The details of the synthesis and characterization of compounds **3a–h** and their spontaneous polymerization are described in Supporting Information. The synthetic intermediates and the solvents were purchased from Sigma-Aldrich (Saint Louis, MO, USA) unless otherwise stated. NMR spectra were recorded with a Bruker (Karlsruhe, Germany) AC200, a Varian (Palo Alto, CA, USA) Mercury-300, a Bruker DRX-400 AVANCE, or a Bruker DRX-600 AVANCE spectrometer in the indicated solvents (TMS as internal standard). The values of the chemical shifts are expressed in ppm and the coupling constants (*J*) in Hz. An Agilent (Santa Clara, CA, USA) 1100 LC/MSD operating with an electrospray source was used in mass spectrometry experiments.

#### 2.2.1. General Procedure for the Preparation of Solutions of Monomers 1a–h in Chloroform or CDCl_3_

A mixture of the appropriate indenol derivative (**3a**–**h**) in chloroform or CDCl_3_ (20 mL/1 mmol of indenol) with a catalytic amount of *p*-toluenesulfonic acid monohydrate (PTSA) (0.2 equivalents) was heated under reflux for 1–2 h and cooled to room temperature, monitoring the reaction by TLC. The reaction mixture was then washed with a saturated solution of NaHCO_3_ and dried over sodium sulfate to afford a stock (about 0.05 M) solution of the corresponding monomer (**1a**–**h**).

*Ethyl 6-Methoxy-3-(2-Methoxyphenyl)-1-methylene-1H-indene-2-carboxylate* (**1a**). This monomer was prepared from indenol **3a** (0.23 g, 0.65 mmol). ^1^H NMR (400 MHz, CDCl_3_): 1.01 (t, *J* = 7.1, 3H), 3.73 (s, 3H), 3.86 (s, 3H), 4.07 (q, *J* = 7.1, 2H), 6.30 (s, 1H), 6.59 (s, 1H), 6.80 (dd, *J* = 8.3, 2.1, 1H), 6.96–7.04 (m, 3H), 7.25 (m, 2H), 7.36 (t, *J* = 7.7, 1H). MS(ESI): *m*/*z* 359 (M + Na^+^).

*Ethyl 6-Methoxy-3-(3-methoxyphenyl)-1-methylene-1H-indene-2-carboxylate* (**1b**). This monomer was prepared from indenol **3b** (0.23 g, 0.65 mmol). ^1^H NMR (400 MHz, CDCl_3_): 1.06 (t, *J* = 7.1, 3H), 3.82 (s, 3H), 3.87 (s, 3H), 4.11 (q, *J* = 7.1, 2H), 6.33 (s, 1H), 6.59 (s, 1H), 6.82 (dd, *J* = 8.4, 2.4, 1H), 6.93–6.99 (m, 3H), 7.13 (d, *J* = 8.4, 1H), 7.25 (s, 1H), 7.34 (t, *J* = 8.2, 1H). MS(ESI): *m*/*z* 359 (M + Na^+^).

*Ethyl 6-Methoxy-3-(4-methoxyphenyl)-1-methylene-1H-indene-2-carboxylate* (**1c**). This monomer was prepared from indenol **3c** (0.62 g, 1.76 mmol). ^1^H NMR (400 MHz, CDCl_3_): 1.12 (t, *J* = 7.1, 3H), 3.86 (s, 3H), 3.87 (s, 3H), 4.15 (q, *J* = 7.1, 2H), 6.30 (s, 1H), 6.55 (s, 1H), 6.83 (dd, *J* = 8.4, 2.3, 1H), 6.97 (d, *J* = 8.8, 2H), 7.16 (d, *J* = 8.4, 1H), 7.25 (d, *J* = 2.4, 1H), 7.37 (d, *J* = 8.8, 2H). MS(ESI): *m*/*z* 359 (M + Na^+^).

*Ethyl 3-(3,4-Dimethoxyphenyl)-6-methoxy-1-methylene-1H-indene-2-carboxylate* (**1d**). This monomer was prepared from indenol **3d** (0.32 g, 0.83 mmol). ^1^H NMR (400 MHz, CDCl_3_): 1.12 (t, *J* = 7.1, 3H), 3.88 (s, 6H), 3.94 (s, 3H), 4.15 (q, *J* = 7.1, 2H), 6.31 (s, 1H), 6.55 (s, 1H), 6.84 (dd, *J* = 8.4, 2.3, 1H), 6.94–7.02 (m, 3H), 7.17 (d, *J* = 8.4, 1H), 7.24 (s, 1H). MS(ESI): *m*/*z* 389 (M + Na^+^).

*Ethyl 6-Methoxy-1-methylene-3-(3,4,5-trimethoxyphenyl)-1H-indene-2-carboxylate* (**1e**). This monomer was prepared from indenol **3e** (0.43 g, 1.04 mmol). ^1^H NMR (400 MHz, CDCl_3_): 1.11 (t, *J* = 7.6, 3H), 3.85 (s, 6H), 3.88 (s, 3H), 3.91 (s, 3H), 4.15 (q, *J* = 7.1, 2H), 6.33 (s, 1H), 6.57 (s, 1H), 6.63 (s, 2H), 6.85 (dd, *J* = 8.4, 2.0, 1H), 7.19 (d, *J* = 8.4, 2H), 7.24 (s, 1H). MS(ESI): *m*/*z* 419 (M + Na^+^).

*Ethyl 6-Methoxy-1-methylene-3-(4-nitrophenyl)-1H-indene-2-carboxylate* (**1f**). This monomer was prepared from indenol **3f** (0.30 g, 0.81 mmol). ^1^H NMR (400 MHz, CDCl_3_): 1.07 (t, *J* = 7.1, 3H), 3.88 (s, 3H), 4.12 (q, *J* = 7.1, 2H), 6.42 (s, 1H), 6.72 (s, 1H), 6.84 (dd, *J* = 8.4, 2.2, 1H), 6.98 (d, *J* = 8.4, 1H), 7.28 (d, *J* = 2.2, 1H), 7.56 (d, *J* = 8.8, 2H), 8.31 (d, *J* = 8.8, 2H). MS(ESI, negative ions): *m*/*z* 701 (2M − H^+^).

*Ethyl 3-(4-Bromophenyl)-6-methoxy-1-methylene-1H-indene-2-carboxylate* (**1g**). This monomer was prepared from indenol **3g** (0.22 g, 0.54 mmol). ^1^H NMR (400 MHz, CDCl_3_): 1.09 (t, *J* = 7.1, 3H), 3.87 (s, 3H), 4.13 (q, *J* = 7.1, 2H), 6.35 (s, 1H), 6.63 (s, 1H), 6.83 (dd, *J* = 8.4, 2.3, 1H), 7.06 (d, *J* = 8.4, 1H), 7.25 (d, *J* = 2.4, 1H), 7.27 (d, *J* = 8.4, 2H), 7.57 (d, *J* = 8.4, 2H). MS(ESI): *m*/*z* 407, 409 (M + Na^+^).

*Ethyl 3-(Furan-3-yl)-6-Methoxy-1-methylene-1H-indene-2-carboxylate* (**1h**). This monomer was prepared from indenol **3h** (0.25 g, 0.80 mmol) and 1 equivalent of PTSA. ^1^H NMR (400 MHz, CDCl_3_): 1.30 (t, *J* = 7.2, 3H), 3.88 (s, 3H), 4.28 (q, *J* = 7.1, 2H), 6.29 (s, 1H), 6.49 (s, 1H), 6.68 (s, 1H), 6.87 (dd, *J* = 8.4, 2.3, 1H), 7.24 (d, *J* = 2.2, 1H), 7.36 (d, *J* = 8.4, 1H), 7.52 (s, 1H) 7.84 (s, 1H). MS (ESI): *m*/*z* 319 (M + Na^+^).

#### 2.1.2. General Procedure for the Preparation of Polybenzofulvene Derivatives Poly-1b–e,g,h by Spontaneous Polymerization.

A stock solution of suitable monomer (**1b**–**e**,**g**,**h**) in chloroform (about 0.05 M) was concentrated under reduced pressure to give a viscous oil, which was dissolved into chloroform (20 mL/mmol of monomer) and newly evaporated. The dissolution/evaporation procedure was repeated from three to nine times, monitoring by TLC the progressive disappearance of the monomer in solution. The final residue was washed with the indicated solvent or dissolved into a small amount of CHCl_3_ and precipitated in the reported bad solvent to give the expected polymer.

*Poly-[Ethyl 6-methoxy-3-(3-methoxyphenyl)-1-methylene-1H-indene-2-carboxylate]* (**Poly-1b**)**.** This polymer was obtained from a stock solution of benzofulvene monomer **1b** in chloroform. The dissolution/evaporation procedure was repeated three times and the final residue was dissolved into a small amount of CHCl_3_ and precipitated with ethanol to give poly-**1b** as a white solid (0.12 g, yield from **3b** 55%). ^1^H NMR spectrum (CDCl_3_, 300 MHz): see Figure S4 in the Supporting Information. ^13^C NMR spectrum (CDCl_3_, 75 MHz): see Figure S27 in the Supporting Information.

*Poly-[Ethyl 6-methoxy-3-(4-methoxyphenyl)-1-methylene-1H-indene-2-carboxylate]* (**Poly-1c**)**.** This polymer was obtained from a stock solution of benzofulvene monomer **1c** in chloroform. The dissolution/evaporation procedure was repeated three times and the final residue was dissolved into a small amount of CHCl_3_ and precipitated with methanol to give poly-**1c** as a white powder (0.41 g, yield from **3c** 69%). ^1^H-NMR spectrum (CDCl_3_, 300 MHz): see Figure S5 in the Supporting Information. ^13^C-NMR spectrum (CDCl_3_, 75 MHz): see Figure S28 in the Supporting Information.

*Poly-[Ethyl 3-(3,4-dimethoxyphenyl)-6-methoxy-1-methylene-1H-indene-2-carboxylate]* (**Poly-1d**). This polymer was obtained from a stock solution of benzofulvene monomer **1d** in chloroform. The dissolution/evaporation procedure was repeated five times and the final residue was dissolved into a small amount of CHCl_3_ and precipitated with ethanol to give poly-**1d** as a pale yellow cottony solid (0.21 g, yield from **3d** 69%). ^1^H NMR spectrum (CDCl_3_, 300 MHz): see Figure S6 in the Supporting Information. ^13^C NMR spectrum (CDCl_3_, 75 MHz): see Figure S29 in the Supporting Information.

*Poly-[Ethyl 6-methoxy-1-methylene-3-(3,4,5-trimethoxyphenyl)-1H-indene-2-carboxylate]* (**Poly-1e**)**.** This polymer was obtained from a stock solution of benzofulvene monomer **1e** in chloroform. The dissolution/evaporation procedure was repeated five times and the final residue was dissolved into a small amount of CHCl_3_ and precipitated with n-hexane to give poly-**1e** as a white cottony solid (0.30 g, yield from **3e** 72%). ^1^H-NMR spectrum (CDCl_3_, 300 MHz): see Figure S7 in the Supporting Information. ^13^C NMR spectrum (CDCl_3_, 75 MHz): see Figure S30 in the Supporting Information.

*Poly-[Ethyl 3-(4-bromophenyl)-6-methoxy-1-methylene-1H-indene-2-carboxylate]* (**Poly-1g**)**.** This polymer was obtained from a stock solution of benzofulvene monomer **1g** in chloroform. The dissolution/evaporation procedure was repeated three times and the final residue was purified by washing with n-hexane to give poly-**1g** as a white solid (0.13 g, yield from **3g** 63%). ^1^H NMR spectrum (CDCl_3_, 300 MHz): see Figure S8 in the Supporting Information. ^13^C NMR spectrum (CDCl_3_, 75 MHz): see Figure S32 in the Supporting Information.

*Poly-[Ethyl 3-(furan-3-yl)-6-methoxy-1-methylene-1H-indene-2-carboxylate]* (**Poly-1h**)**.** This polymer was obtained from a stock solution of benzofulvene monomer **1h** in chloroform. The dissolution/evaporation procedure was repeated nine times and the final residue was dissolved into a small amount of CHCl_3_ and precipitated with *n*-hexane to give poly-**1h** as a beige powder (0.13 g, yield from **3h** 55%). ^1^H-NMR spectrum (CDCl_3_, 400 MHz): see Figure S9 in the Supporting Information. ^13^C NMR spectrum (CDCl_3_, 75 MHz): see Figure S33 in the Supporting Information.

*Poly-[Ethyl 6-methoxy-3-(2-methoxyphenyl)-1-methylene-1H-indene-2-carboxylate]* (**Poly-1a**)**.** The stock solution of benzofulvene monomer **1a** in chloroform (about 0.05 M) was concentrated under reduced pressure to give a viscous oil, which was dissolved into chloroform (20 mL/mmol of monomer) and newly evaporated. The dissolution/evaporation procedure was repeated several times without the complete disappearance of the monomer (the progress of the polymerization process was monitored by TLC). The final residue was dissolved into a small amount of CHCl_3_ and precipitated with ethanol to give poly-**1a** as a white solid (0.070 g, yield from **3a** 32%). ^1^H NMR spectrum (CDCl_3_, 300 MHz): see Figure S10 in the Supporting Information. ^13^C NMR spectrum (CDCl_3_, 75 MHz): see Figure S26 in the Supporting Information.

*Poly-[Ethyl 6-methoxy-1-methylene-3-(4-nitrophenyl)-1H-indene-2-carboxylate]* (**Poly-1f**). The stock solution of benzofulvene monomer **1f** in chloroform (about 0.05 M) was concentrated under reduced pressure to give a viscous brown oil. The oil was suspended into chloroform (10.0 mL/mmol of monomer) to obtain a yellow precipitate. The precipitate was collected by filtration, dissolved in a small amount of dichloromethane, and newly precipitated in ethanol to obtain poly-**1e** as a yellow powder (0.22 g, yield from **3f** 77%).^1^H-NMR spectrum (CDCl_3_, 300 MHz): see Figure S11 in the Supporting Information. ^13^C-NMR spectrum (CD_2_Cl_2_, 75 MHz): see Figure S31 in the Supporting Information.

### 2.2. SEC-MALS

The MWD characterization of the polymers was performed by a Wyatt MALS light scattering photometer (Santa Barbara, CA, USA) on-line to a Waters SEC chromatographic system (Milford, MA, USA). The SEC-MALS system and the corresponding experimental conditions were identical to those used in our previous studies [30,32] and are not reported in detail here.

### 2.3. X-ray Crystallography

Single crystals of **2a**–**f**,**h**, **3e**–**g**, **6c**–**f**,**h**, **7**, and **9** were submitted to X-ray data collection on an Oxford-Diffraction Xcalibur Sapphire 3 (Santa Clara, CA, USA) diffractometer with a graphite monochromated Mo-*K*α radiation (λ = 0.71073 Å) at 293 K. The structures were solved by direct methods implemented in SHELXS-2013 program [55]. The refinements were carried out by full-matrix anisotropic least-squares on F^2^ for all reflections for non-H atoms by means of the SHELXL-2018 program [56]. Crystallographic data (excluding structure factors) for the structure in this paper have been deposited with the Cambridge Crystallographic Data Centre as supplementary publications no. CCDC 1816328 (**2a**), 1816327 (**2b**), 1816303 (**2c**, polymorph A), 1816324 (**2c**, polymorph B), 1816305 (**2d**), 1816329 (**2e**), 1816304 (**2f**), 1816323 (**2h**), 1816054 (**3e**), 1816053 (**3f**), 1816055 (**3g**), 1816312 (**6c**), 1816315 (**6d**), 1816331 (**6e**), 1816313 (**6f**), 1816314 (**6h**), 1816333 (**7**), and 1816322 (**9**). Copies of the data can be obtained, free of charge, on application to CCDC, 12 Union Road, Cambridge CB2 1EZ, UK; (fax: +44(0)-1223-336-033; or e-mail: deposit@ccdc.cam.ac.uk).

## 3. Results

### 3.1. Design, Synthesis, and Spontaneous Polymerization of Benzofulvene Derivates ***1a**–**h***

**Figure 2:**
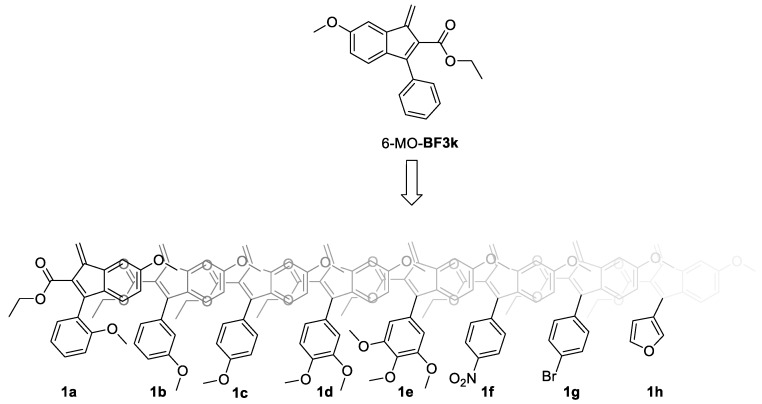
Design of the new benzofulvene monomers **1a**–**h**.

The benzofulvene monomers were designed starting from the previously published methoxyderivative 6-MO-**BF3k** (Figure 2) [2,49].

The pendant phenyl ring of 6-MO-**BF3k** was modified by introducing one or more electron donor methoxy groups in *ortho*, *meta*, and *para* positions (compounds **1a**–**e**), an electron withdrawing nitro group (**1f**), or a bromine substituent (**1g**) in *para* position, and finally, by replacing the pendant phenyl with a furan ring (**1h**).

**Scheme 1:**
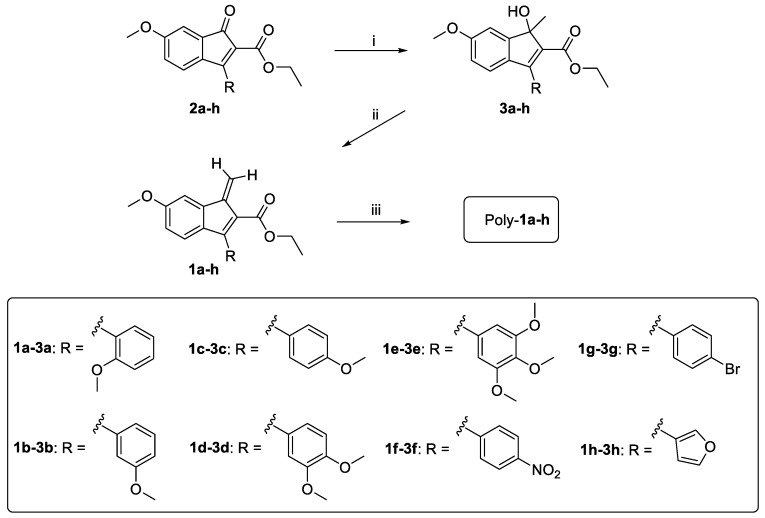
Synthesis and spontaneous polymerization of benzofulvene monomers **1a**–**h. Reagents**: (i) Al(CH_3_)_3_ (or CH_3_MgBr for compound **2h**), CH_2_Cl_2_; (ii) PTSA, CHCl_3_; (iii) solvent removal.

Benzofulvene derivatives **1a**–**h** were synthesized by following the synthetic pathway developed in our laboratories and shown in Scheme 1.

Indenone derivatives **2a**–**h** were reacted with trimethylaluminium (or methyl magnesium bromide for **2h**) to furnish the required indenol derivatives **3a**–**h**, which were dehydrated in the presence of *p*-toluenesulfonic acid (PTSA) to afford the corresponding monomers **1a**–**h**. Finally, the tendency to the spontaneous polymerization of the benzofulvene monomers was evaluated in terms of the formation of polymeric materials (i.e., poly-**1a**–**h**) as a consequence of the solvent removal under reduced pressure without the deliberate addition of catalysts or initiators.

**Scheme 2:**
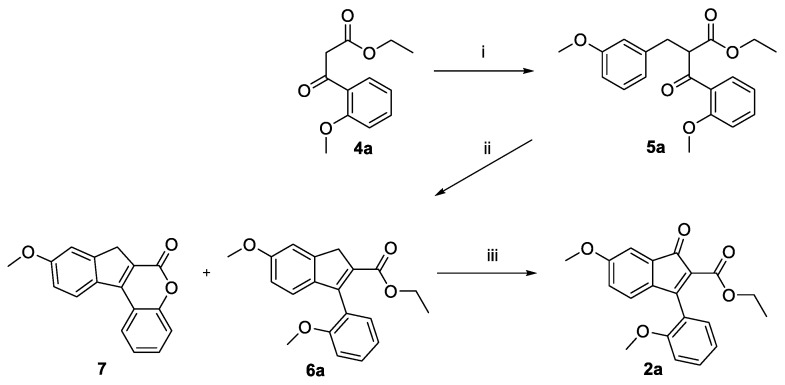
Synthesis of indenone derivative **2a**. **Reagents**: (i) 3-methoxybenzyl chloride, K_2_CO_3_, NaI, DMF; (ii) PPA; (iii) SeO_2_, 1,4-dioxane.

Indenone **2a** was prepared as described in Scheme 2.

The reaction of 3-methoxybenzyl chloride with ethyl (2-methoxybenzoyl)acetate (**4a**) provided compound **5a**, which was cyclized in polyphosphoric acid (PPA) to obtain indene derivative **6a** together with small amounts of compound **7**, which could be considered the conformationally locked analogue of **6a** at the pendant phenyl. Finally, indenone derivative **2a** was obtained by selective oxidation of **6a** with selenium dioxide.

**Scheme 3:**
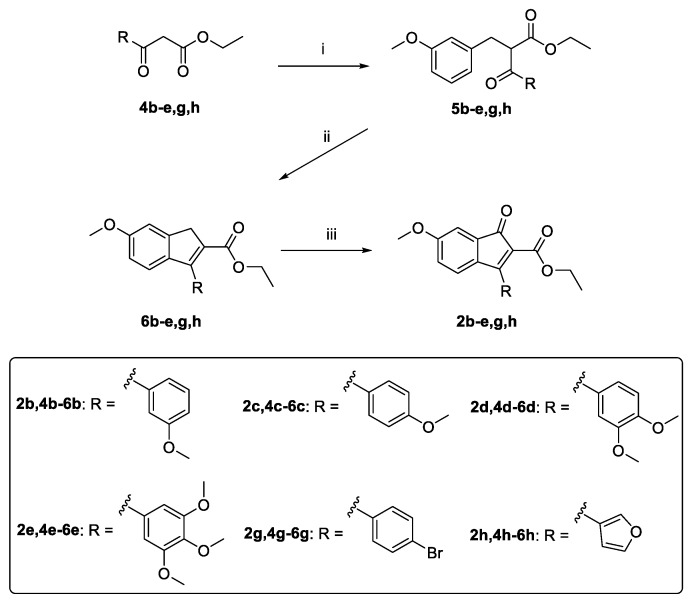
Synthesis of indenone derivatives **2b**–**e**,**g**,**h**. **Reagents**: (i) 3-methoxybenzyl chloride, K_2_CO_3_, NaI, DMF; (ii) PPA; (iii) SeO_2_, 1,4-dioxane.

Similarly, indenones **2b**–**e**,**g**,**h** were prepared by using 3-methoxybenzyl chloride and the appropriate β-ketoester **4b**–**e**,**g**,**h** as starting materials (Scheme 3).

**Scheme 4:**
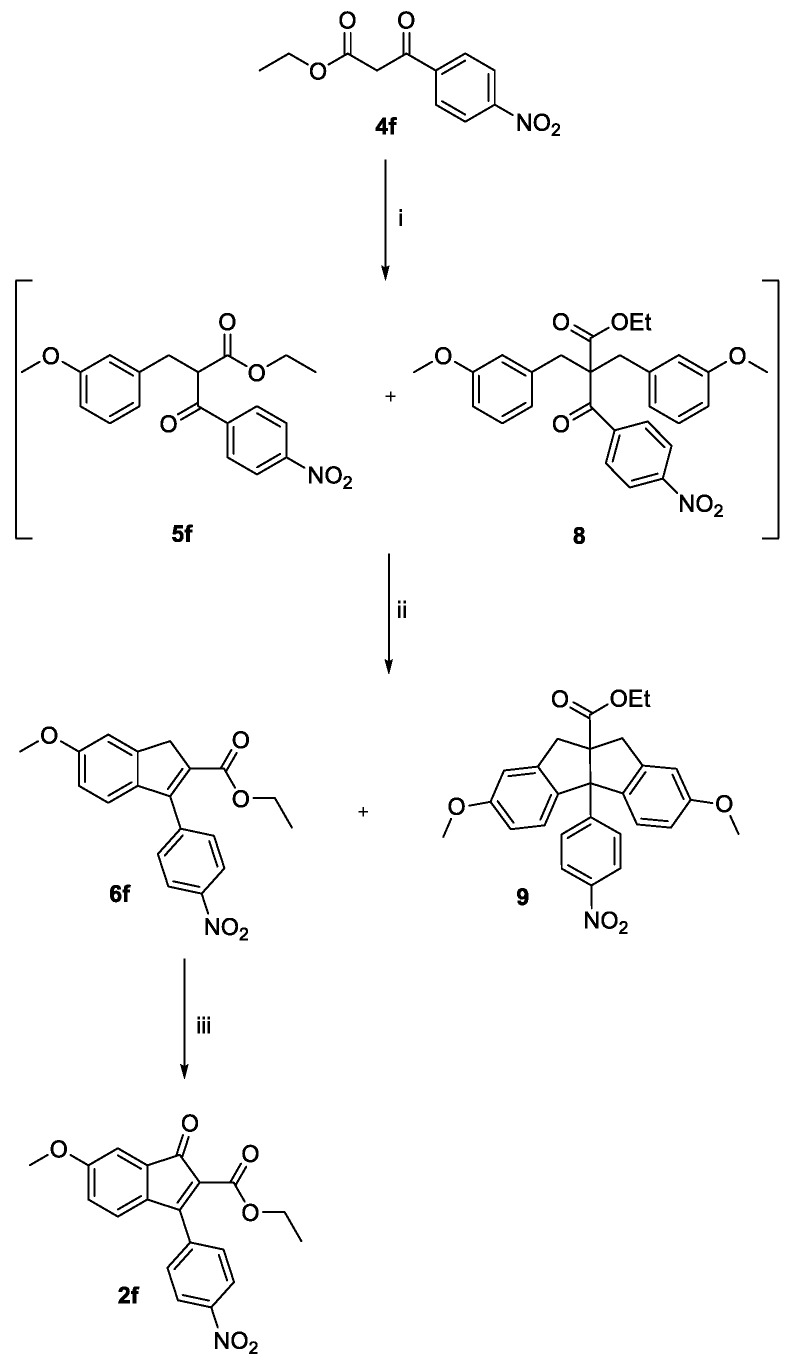
Synthesis of indenone derivative **2f**. **Reagents**: (i) 3-methoxybenzyl chloride, K_2_CO_3_, NaI, DMF; (ii) PPA; (iii) SeO_2_, 1,4-dioxane.

On the other hand, some little discrepancies were found during the synthesis of indenone **2f** (Scheme 4). The reaction of 3-methoxy benzyl chloride with ethyl (4-nitrobenzoyl)acetate (**4f**) gave a mixture of monoadduct **5f** and biadduct **8**. The subsequent cyclization of this mixture with PPA furnished the cyclized derivatives **6f** and **9**, which were separated by chromatography and characterized by X-ray crystallography (Figures S17 and S18). Finally, the selective oxidation of compound **6f** afforded the expected indenone **2f**.

The synthetic intermediates **6a**–**h** were characterized as the models of monomeric units of the relevant polymers.

### 3.2. Properties of Polybenzofulvene Derivatives Poly-***1a**–**h***

The standard procedure for the spontaneous polymerization developed over the years in our laboratory was applied to the monomers **1a**–**h**. This procedure required the evaporation at reduced pressure of the solvent (usually chloroform) from a stock solution of monomer (see Experimental Section and Supplementary Materials for the details) to obtain a thick oil, which was solubilized in the same good solvent and concentrated again. The dissolution-concentration procedure was repeatedly applied several (usually 3–5) times, monitoring each step by TLC to evaluate the progressive disappearance of the monomer and the resulting formation of the polymer. Almost all the newly synthesized monomers showed a good tendency towards spontaneous polymerization leading to the substantial disappearance of the monomer from the solution, with the exception of monomers **1a**,**f**, which showed apparently anomalous behaviors. In particular, the spontaneous polymerization of monomer **1a**, bearing the methoxy group in position 2′ of the pendant phenyl, was proven to be difficult, with relatively high amounts of the monomer remaining in solution even after the application of the concentration/dissolution procedure up to nine times. On the contrary, monomer **1f** showed a strong tendency toward spontaneous polymerization, which could be considered almost complete immediately after the first concentration. Thus, poly-**1f** was obtained by applying the concentration of the stock solution only one time because, together with the virtual absence of monomer after the first evaporation of the solvent, the resulting polymeric material was demonstrated to be virtually insoluble in chloroform.

Poly-**1a**–**h** were purified by the dropwise addition of the polymer solution in a good solvent (i.e., chloroform or dichloromethane) into a suitable excess amount of a bad solvent, such as ethanol or hexane, leading to fine precipitates, which were easily dried to fine powders.

Owing to their lipophilic nature, poly-**1a**–**h** were insoluble in water, ethanol, and methanol, while they were readily soluble in the most common organic solvents such as chloroform, dichloromethane, diethyl ether, and tetrahydrofuran, with the exception of poly-**1f**, which was readily soluble in dichloromethane but quite surprisingly almost insoluble in most the other organic solvents, suggesting the existence of strong intra- and interchain interactions. 

### 3.3. Molecular Weight Distribution of Polybenzofulvene Derivatives Poly-***1a**–**h***

The molecular weight distribution (MWD) and also some conformation data of the newly synthesized polybenzofulvene derivatives were characterized by a multidetector size exclusion chromatography (SEC or GPC) system. The multidetector SEC system used two on-line detectors: (1) multiangle laser light scattering (MALS) and (2) differential refractometer (DRI) as concentration detectors. The SEC-MALS experimental conditions were: tetrahydrofuran (THF) as mobile phase; 2 PLgel Mixed C columns from Polymer Laboratories; 35 °C of temperature; 0.8 mL/min of flow rate; about 1 mg/mL of sample concentration. Molecular weight values from an SEC-MALS system are absolute without use of an external relative calibration. Table 1 summarizes the most important SEC-MALS results for polybenzofulvene derivatives. In particular, Table 1 reports: molecular weight of chromatogram peak (*M*_p_); molecular weight average (*M*_w_); polydispersity index (PDI); average dimension of macromolecules or radius of gyration (*R*_g_); intercept (*K*); and slope (α) of the conformation plot (*R*_g_ = *K*·*M*^α^). Specifically, the slope (α) of the conformation plot furnishes important information on the stiffness of the polybenzofulvene derivatives macromolecular chain.

The analysis of the results reported in Table 1 suggested that the spontaneous polymerization process of the benzofulvene derivatives appeared to be compatible with the presence of a large variety of substituent in the pendant phenyl ring attached to position 3 of the indene nucleus. However, a modulation of the macromolecular features of the corresponding polybenzofulvene derivatives was observed to be related to the steric features of the substituent and to their positions. In particular, the presence of one methoxy group in the positions 3′ or 4′ appeared to produce negligible effects, whereas the presence of the same substituent in position 2′ led to a decrease of about one order of magnitude in the molecular weight values (compare poly-**1a** with poly-**1b**,**c**). The decoration of the pendant phenyl ring with two or three methoxy groups in positions 3′, 4′, and 5′ seems to be well tolerated in the polymerization process leading to poly-**1d**,**e**. Curiously, the presence of the bromine atom in position 4′ led to a polymer (poly-**1g**) showing an ultra-high molecular weight, while the replacement of the phenyl ring with the furan ring provided a polymer (poly-**1h**) showing a low molecular weight.

As far as the stiffness of macromolecules is concerned, the newly synthesized polybenzofulvene derivatives showed quite different behaviours. The slope α of the conformational plot, for the most of these polybenzofulvene derivatives, ranged between 0.48 and 0.61 (poly-**1b**–**e**,**g**). Such slope α values are typical of random coil polymers in an ideal theta solvent or in a good solvent. An exception is represented by poly-**1h**, where the slope α value is 0.35, which suggests more compact conformation and/or bad solvent and/or presence of long chain branching (LCB). In fact, polymers with low α values (<0.5) possess a more compact, dense structure and/or low solubility and/or meaningful presence of LCB.

### 3.4. NMR Studies on Polybenzofulvene Derivatives Poly-***1a**–**h***

**Figure 3:**
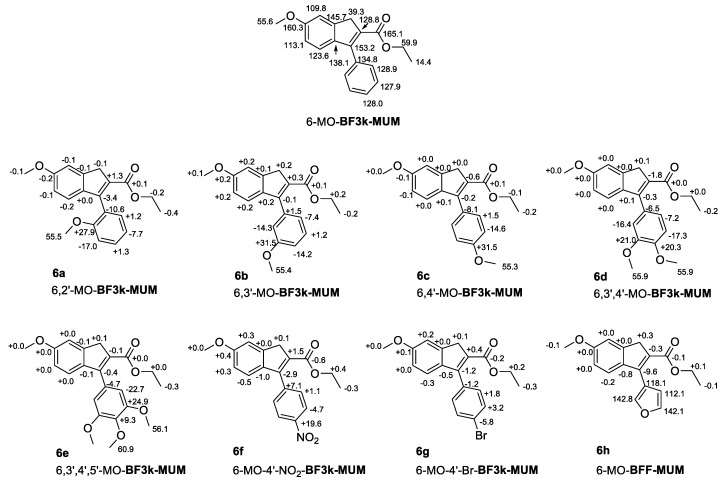
Effects of the pendant ring manipulation on the ^13^C NMR chemical shift values of the model compounds **6a**–**h** with respect to 6-MO-**BF3k-MUM**.

The macromolecular structures of poly-**1a**–**h** were investigated by means of different NMR techniques. As expected, the ^1^H NMR spectra of these polymers were characterized by broad signals, whereas the corresponding ^13^C NMR spectra were more resolved although quite complex and constituted by partially merging signals (see Figures in SI). Therefore, to better understand the NMR spectra, indene derivatives **6a**–**h** were studied as the models of the monomeric units of the polymers with the assumption that the vinyl (1,2) mechanism was operative in the spontaneous polymerization of monomers **1a**–**h**. Thus, in the first step of the study, the ^1^H and ^13^C NMR spectra of compounds **6a**–**h** were fully assigned by means of homonuclear ^1^H-^1^H COSY [57], ^1^H-^1^H-NOESY [58] and heteronuclear ^1^H-^13^C-HSQC [59], ^1^H-^13^C-HMBC [60] 2D correlation experiments. The assigned ^13^C spectra of the model compounds **6a**–**h** were checked in a comparative study with respect to the model compound 6-MO-**BF3k**-**MUM** [2] (Figure 3) in order to evaluate the effects of the structural manipulations on the electron distribution in the molecular orbital of the monomeric units.

These comparisons demonstrated that the manipulations of the pendant phenyl through the introduction of different (i.e., electron donor or withdrawing) groups or its replacement with the furan ring caused only negligible changes at the level of the indene carbons with the exception of C-3. In fact, C-3 chemical shift values were significantly shifted in compounds **6a** (−3.4 ppm), **6f** (−2.9 ppm), **6g** (−1.2 ppm), and **6h** (−9.6 ppm) with respect to the corresponding signal of 6-MO-**BF3k-MUM**. On the contrary, the substituents implemented on the pendant phenyl ring produced significant changes in chemical shift of all the signals of the carbon atoms belonging to the pendant phenyl portion in each model compound. This observation suggested the existence of little overlap between the π-orbitals of the two conjugate aromatic moieties, namely the pendant phenyl group and the indene moiety.

As expected, the observed changes were compatible with inductive electron withdrawing and electron donating effects. Among them, the value of C-1′ signals substantially moved upfield in the presence of the methoxy substituent in *ortho* (2′; −10.6 ppm in **6a**) or *para* positions (4′; −8.1 ppm in **6c**) and it moved only slightly when a bromine was present in the *para* position (−1.2 ppm in **6g**). On the other hand, the chemical shift value of C-1′ signal moved slightly downfield when the methoxy group occupied the *meta* position (+1.5 ppm in **6b**), but it significantly did this when a nitro group was present in *para* position (+7.1 ppm in **6f**). In the cases of the double and triple methoxy substitution on the pendant phenyl, the value of C-1′ chemical shift was the result of the *meta* and *para* methoxy effects (−6.5 and −4.7 ppm, in **6d** and **6e**, respectively). Finally, for comparative purposes, the chemical shift of the C-1′ signal in compound **6h** showed a considerable upfield shift, being 118.1 ppm.

The chemical shift values of C-1′ and C-3 signals in the monomeric unit models were used to discriminate between 1,2 or 1,4 polymerization mechanisms. In fact, in the case of 1,2 (vinyl type) polymerization mechanism of monomers **1a**–**h**, carbon C-1′ was bonded to unsaturated C-3 atoms as in the case of **6a**–**h** models, whereas in a polymeric structure with 1,4 chaining, the double bond is shifted and C-1′ is linked to a saturated C-3 atom.

**Figure 4:**
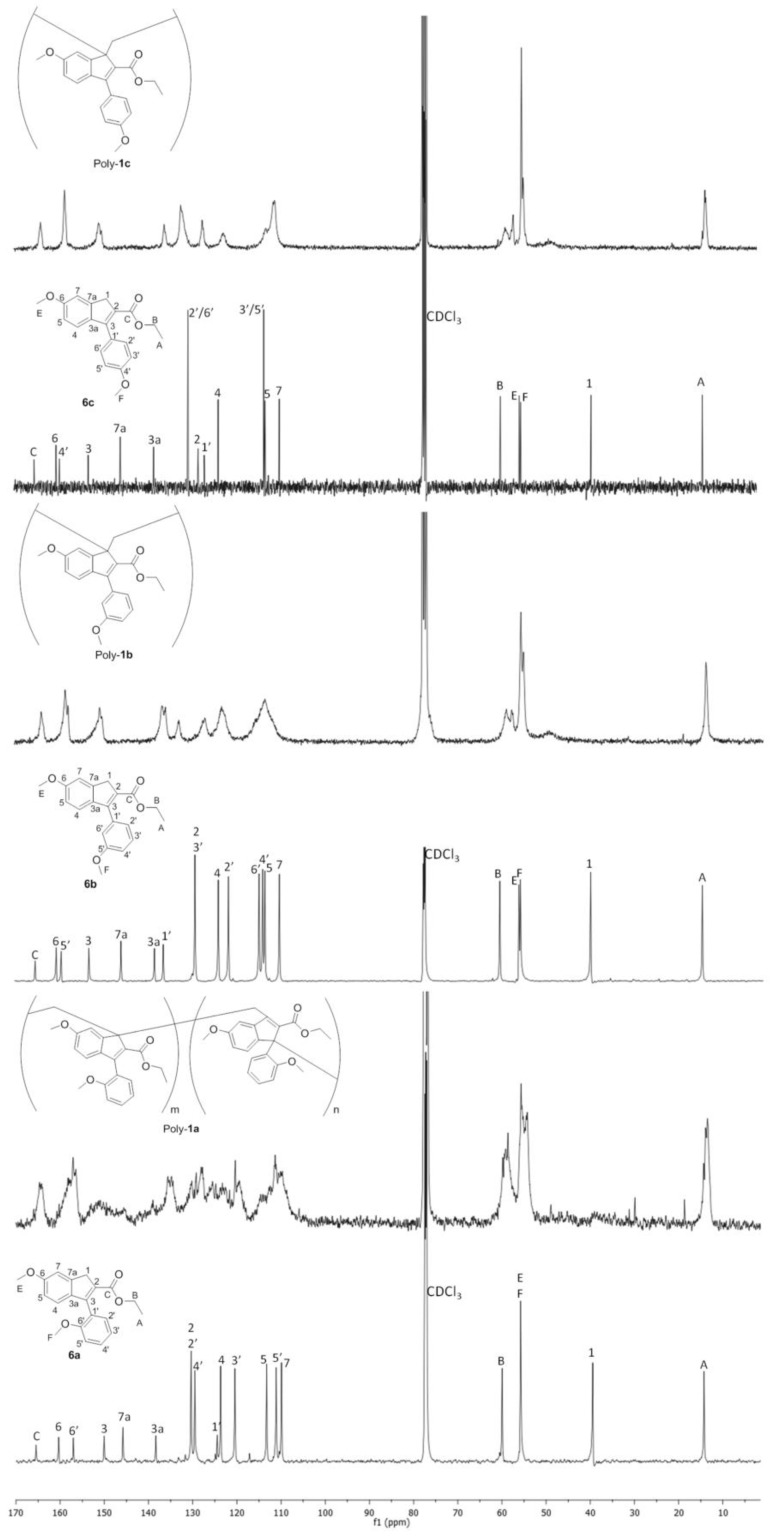
^13^C NMR spectra (CDCl_3_) of the polymers poly-**1a**–**c** in comparison with the corresponding monomeric unit models **6a**–**c**.

**Figure 5:**
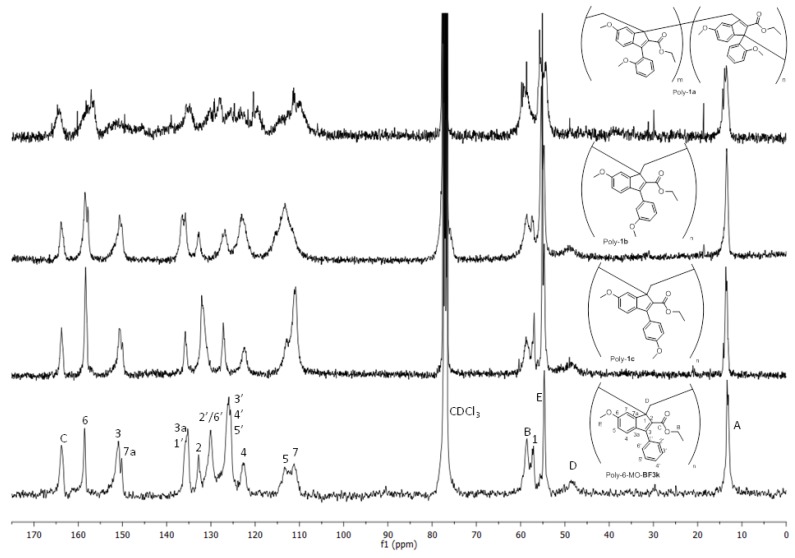
Comparison of the ^13^C NMR spectra (CDCl_3_) of poly-**1a**–**c** with that of the previously reported poly-6-MO-**BF3k** [49].

**Figure 6:**
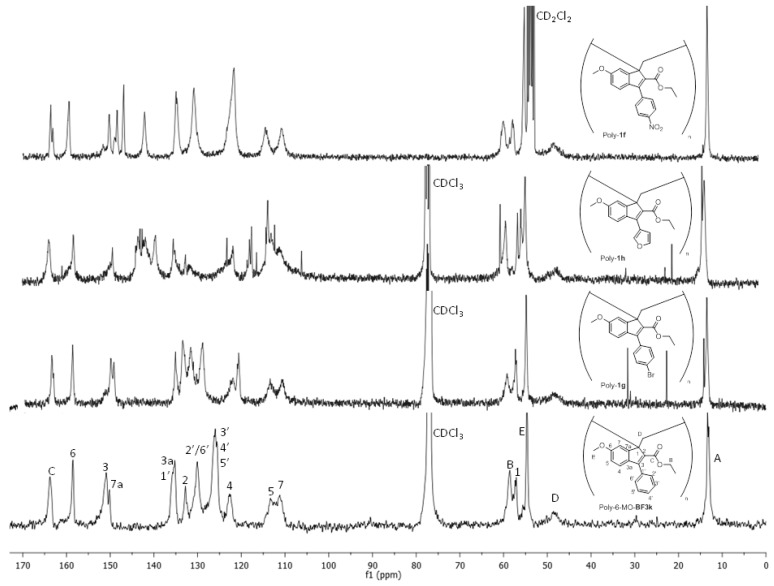
Comparison of the ^13^C NMR spectrum of the previously reported poly-6-MO-**BF3k** with those of the new polymers poly-**1g**,**h** (CDCl_3_) and poly-**1f** (CD_2_Cl_2_).

The ^1^H NMR spectra of poly-**1a**–**h** were similar to those shown by the other members of this class of polymers and provided only little structural information. They were characterized by broad signals, which appeared to be slightly shifted upfield with respect to the signals of the corresponding protons in the monomer unit models. On the other hand, the ^13^C NMR spectra were characterized by more defined signal patterns and could be used to grasp structural information on the 1,2 or 1,4 linkage between the repeating units (Figure 4, Figure 5 and Figure 6).

The comparison of all the ^13^C NMR spectra of the synthesized polymers led to the recognition of some diagnostic signals that could provide information on the type of linkage within the polybenzofulvene backbone. The first diagnostic signal taken into consideration was the carbon of the methylene bridge (C–D signal), which appeared as a two-component broad peak at 48–50 ppm in the previously synthesized polymers showing the 1,2 enchainment. By the contrast, in some benzofulvene polymers bearing a 1,4 enchainment, the signal attributed to the carbon C–D was shifted at about 38–40 ppm [30,49].

All the newly synthesized polymers (poly-**1a**–**h**) showed the presence of the broad signal around 48–50 ppm, compatible with a 1,2 vinyl enchainment, but the poly-**1a** spectrum showed, together with the very broad signal at 49.1 ppm, also another signal of equal intensity at about 38.7 ppm that suggested the concomitant presence of polymeric sequences showing 1,4 enchainment.

Moreover, the comparison of the ^13^C NMR spectra of poly-**1b**–**h** with those of the corresponding model compounds **6b**–**h** (see Figures in SI) showed virtually identical chemical shift values and signal patterns of the peaks attributed to C-1′ and C-3 that should be affected by the potentially competing 1,4 polymerization. These findings appeared to support the retention of the spontaneous 1,2 polymerization mechanism in poly-**1b**–**h**.

On the other hand, the spectrum of the poly-**1a** was not perfectly matching with the spectrum of the monomeric unit model **6a** representative of 1,2 linkage in an ideal polymer. In particular, the ^13^C-NMR spectrum of poly-**1a** showed, in correspondence of the signal attributed to C-3 of **6a**, a very broad and confused peak of low intensity. A similar behavior was found also for the signal attributed to C-1′ carbon. Therefore, the ^13^C-NMR spectrum of poly-**1a** suggested that the material obtained was structurally heterogeneous and probably the polybenzofulvene backbone showed the presence of 1,4 sequences along with 1,2 ones. Moreover, the difficult spontaneous polymerization of **1a** suggested a key role of the hydrogen in 2′-position in the establishment of productive interactions between the monomers, which are assumed to be the basis of the spontaneous polymerization and could be hindered in **1a** by the presence of the methoxy group.

### 3.5. Single Crystal X-ray Diffraction Studies on the Synthetic Intermediates of Polybenzofulvene Derivatives Poly-***1a**–**h***

On the basis of the observation that only the monomers capable of interacting with the benzofulvene molecules are included in the corresponding polymer, we developed the concept of “affinity polymerization”, based on a two-step mechanism [29,50]. In particular, we assumed that in the molecular recognition step, the benzofulvene derivatives could form columnar aggregates in concentrated solutions and in the solid state. The aggregates are capable of organizing their architecture on the basis of their short-range interactions (i.e., specific aromatic interactions) and, when the intermonomer distance becomes compatible with the formation of covalent bonds among the monomers, the spontaneous polymerization starts and progresses rapidly as a domino reaction (polymerization step).

**Figure 7:**
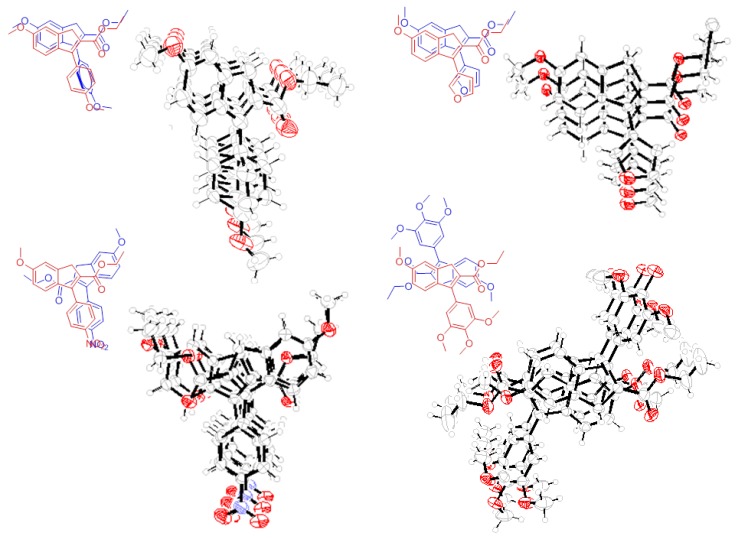
Crystal packing of indene derivatives **6c** (**top left**), **6h** (**top right**), **6f** (**bottom left**), and **6e** (**bottom right**).

We also assumed that the pendant phenyl ring of 3-phenylbenzofulvene derivatives could play a pivotal role in aggregation, structure organization, polymerization, and perhaps also in determining backbone features, such as conformational preferences, stiffness, and stability, through the establishment of specific aromatic (i.e., T-shaped) interactions (Figure 7).

Unfortunately, the tendency toward spontaneous polymerization of benzofulvene monomers precluded the study of their interaction in the solid state. Thus, in order to obtain information on the molecular recognition process preluding the spontaneous polymerization, the solid state interactions of synthetic intermediates **2a**–**f**,**h**, **3e**–**g**, and **6c**–**f**,**h** were characterized by X-ray diffraction studies and were involved in a comparative analysis together with compounds **2g** and **6g** previously reported in [41].

The analysis of the crystal structures highlights the presence of columnar aggregates in different samples. In particular, in the crystals of the model compound **6c**, the molecules are arranged to form columnar aggregates with their indene moieties in a parallel orientation (with the ester groups in the same orientation) and the pendant phenyl rings establishing specific aromatic T-shaped interactions. The same type of aggregation in solid state is also present in one of the two crystalline forms of the corresponding indenone **2c** with dihedral angles between the two least squares planes of 86.01°. A similar arrangement of the molecules is also present in the crystal of the monomer unit model **6h** bearing the furan ring with a face-to-face orientation between the molecules of the columnar aggregates, with an interplanar distance of 3.662 Å. The face-to-face interaction of the pendant ring is also found in the model of the monomeric unit **6f**, which shows columnar aggregates organized with an alternate orientation of the indene portions. Curiously, in the model compounds **6e** and **6g**, the molecules organize antiparallel columnar aggregates in which the indene portions not only alternate the orientation of the ester group on the horizontal axis (similar to **6f**) but also the position of the whole molecule on the vertical axis. This antiparallel orientation is also maintained in the corresponding indenone derivatives **2e**–**g** and also in the indenone **2b**.

In all these aggregates, the aromatic moieties of the synthetic intermediates are capable of establishing a large variety of short-range interactions and a particular role seems to be played by the hydrogen in position 2′. In fact, in the different structures, H-2′ seems to interact with an electron-rich system of the adjacent molecules, such as the pendant aromatic ring by T-shaped interactions, the carbonyl oxygen atoms (of the ester group, in some indene derivatives, or of the ketone moiety in some indenone derivatives), ethereal oxygens, but also with the fused benzene ring. This interaction and the organization of the molecules in the solid state seems to be absent in the crystal of the indenone **2a** (the synthetic intermediate of poly-**1a** producing crystals suitable for crystallographic studies), which shows the position 2′ occupied by the methoxyl group. These observations confirm the importance of the hydrogen atom in this position in the molecular recognition process before polymerization and in defining the final macromolecular structure.

## 4. Conclusions

Differently from other fulvene derivatives (i.e., **DBF** and **BF**), many 3-phenylbenzofulvene derivatives were found to polymerize spontaneously, in the apparent absence of catalysts or initiators, by solvent removal leading the corresponding polymers without side reactions or byproducts. This spontaneous polymerization process was previously investigated by synthesizing benzofulvene monomers bearing different substituents on the indene moiety. These studies highlighted the key role played by the substituent in position 2, where bulky groups depressed spontaneous polymerization, while very small groups led to the formation of materials showing nonhomogeneous structures. The electron-withdrawing ethyl ester group in this position plays a fundamental role for the spontaneous formation of polybenzofulvene derivatives showing preferential (1,2) vinyl structure. Nevertheless, the phenyl ring in position 3 of the indene nucleus seems to be crucial for the spontaneous polymerization of our benzofulvene monomers. In fact, we assumed that the pendant phenyl ring can establish productive π-stacking interactions between the monomers in solution and between the repeating units in the polymers [29].

Eight new 3-substituted benzofulvene monomers **1a**–**h** were synthesized in order to investigate the effect on the spontaneous polymerization of electron-donor or -withdrawing groups on the pendant phenyl ring or its replacement with the furan ring. All monomers polymerized spontaneously by solvent removal, in the apparent absence of catalysts or initiators, to give the corresponding polymers. However, the spontaneous polymerization of monomer **1a**, bearing a methoxy group in position 2′, was found to be difficult, leading to relatively low amounts of a polymeric material and substantial amounts of the monomer without the appearance of other side products. The results of SEC-MALS studies suggest that the polymeric material obtained from monomer **1a** was characterized by a low molecular weight (*M*_w_ = 38.8 Kg/mol), while the other 3-phenyl substituted benzofulvene monomers furnished polymers showing molecular weights ranging from high (*M*_w_ = 191 Kg/mol for poly-**1d**) to ultra-high (*M*_w_ = 2269 Kg/mol for poly-**1g**). The presence of a furan ring in position 3 of the indene nucleus (as in monomer **1h**) is compatible with the effective spontaneous polymerization of the benzofulvene monomer, although the resulting polymer (poly-**1h**) was characterized by a low molecular weight (*M*_w_ = 59.3 Kg/mol).

The macromolecular structures of the new polybenzofulvene derivatives were investigated by NMR spectroscopy by comparing their ^13^C NMR spectra with those of the corresponding models of the monomeric units **6a**–**h**. Firstly, a careful comparison of the chemical shift values of the carbon atoms in the model **6a**–**h** with respect to the naked model 6-MO-**BF3k**-MUM demonstrated that the substituents on the phenyl ring affect the electronic distribution in the monomeric unit only at the level of this latter moiety, while the indene nucleus was marginally involved, with the exception of the carbon-3.

The comparison of the ^13^C NMR spectra of the polymers with those of the corresponding monomer unit models and with the one of the parent polymer poly-6-MO-**BF3k** suggests that spontaneous vinyl (1,2) polymerization is preferred in all synthesized polymers with exception of poly-**1a**. In fact, the ^13^C-NMR spectrum of this polymer shows very broad signals in place of the diagnostic peaks, suggesting a heterogeneous structure with the contemporary presence of 1,2 and 1,4 sequences along the backbone. The difficult spontaneous polymerization of monomer **1a** taken together with the low polymerization yield, the inhomogeneous structure, and the low molecular weight suggested the importance of the hydrogen in 2′-position for productive interactions in the aggregation of the monomers (recognition step) and in the consequent spontaneous polymerization. Specific aromatic (i.e., T-shaped) interactions involving the pendant phenyl could play a crucial role in governing the behavior of our 3-phenylbenzofulvene derivatives, as suggested by the single crystal X-ray diffraction studies on the synthetic intermediates of polybenzofulvene derivatives poly-**1a**–**h**. These interactions could be hindered in **1a** by the presence of the methoxy group, while they continue to be permitted also by replacing the phenyl ring with the furan ring of **1h**.

Finally, this study opens the way to modify the pendant ring in position 3 of the indene nucleus to design new polybenzofulvene derivatives differently decorated in a portion of the monomeric unit, which should not suffer from overcrowding problems because of the distance from the polymerization center (in the monomer) and from the backbone in the polymer.

## Figures and Tables

**Table 1:** Macromolecular features of the newly synthesized poly-**1a**–**h** compared with those of the previously reported poly-**BF3k** and poly-6-MO-**BF3k**.

Polymer	*M*_p_ (kg/mol)	*M*_w_ (kg/mol)	*PDI*	*R*_g_ (nm)	*K* (nm)	α
poly-**1a**	44.1	38.8	1.5	7.7	0.0104	0.61
poly-**1b**	365	400	1.9	22.4	0.0261	0.51
poly-**1c**	754	782	2.0	32.3	0.0117	0.56
poly-**1d**	181	191	1.8	13.0	0.0212	0.51
poly-**1e**	748	831	3.0	31.0	0.0253	0.50
poly-**1f**	nd ^a^	nd ^a^	nd ^a^	nd ^a^	nd ^a^	nd ^a^
poly-**1g**	2950	2269	2.8	58.5	0.0433	0.48
poly-**1h**	66.8	59.3	2.4	8.7	0.1670	0.35
poly-**BF3k**	1900	1506	3.4	49.9	0.0066	0.60
poly-6-MO-**BF3k**	312	347	4.3	19.4	0.0057	0.61

^a^ nd: not determined.

## Supplementary Materials

Experimental details for the synthesis and the characterization of poly-**1a**–**h** and their intermediates are reported. The supplementary materials are available online at http://www.mdpi.com/2073-4360/10/7/752/s1.
