# Supplementary material for: Structural Manipulation of the Conjugated Phenyl Moiety in 3-Phenylbenzofulvene Monomers: Effects on Spontaneous Polymerization
Source: Polymers (Basel). 2018 Jul 7;10(7):752. doi: 10.3390/polym10070752 (PMC6403996; doi:10.3390/polym10070752)
Supplement: Supplementary file 1 [file polymers-10-00752-s001.pdf]

Supplementary Materials for

Structural manipulation of the conjugated phenyl moiety in 3-phenylbenzofulvene monomers: effects on spontaneous polymerization

Marco Paolino,*[,a] Giorgio Grisci,[a] Annalisa Reale,[a] Vincenzo Razzano,[a] Germano Giuliani,[a] Alessandro Donati,[a] Raniero Mendichi,[b] Daniele Piovani,[b] Antonella C. Boccia,[b] Alessandro Grillo,[a] Gianluca Giorgi,[a] Andrea Cappelli.*[,a]

[a]Dipartimento di Biotecnologie, Chimica e Farmacia (Dipartimento di Eccellenza 2018-2022), Università degli Studi di Siena, Via Aldo Moro 2, 53100 Siena, Italy

[b]Istituto per lo Studio delle Macromolecole (CNR), Via E. Bassini 15, 20133 Milano, Italy

Synthesis.

The synthesis of compounds **2g**, **5g**, and **6g** are reported in ref 1. Melting points were determined in open capillaries in a Gallenkamp apparatus and are uncorrected. NMR spectra were recorded with a Bruker AC200, a Varian Mercury-300, a Bruker DRX-400 AVANCE, or a Bruker DRX-600 AVANCE spectrometer in the indicated solvents (TMS as internal standard): the values of the chemical shifts are expressed in ppm and the coupling constants (*J*) in Hz. An Agilent 1100 LC/MSD operating with an electrospray source was used in mass spectrometry experiments.

General procedure for the synthesis of compounds 3a-g.

To a solution of the appropriate indenone derivative (**2a-g**, 1 equivalent) in dichloromethane (20 mL) was added a 2M solution of $Al(CH_3)_3$ in toluene (1 equivalent). The reaction mixture was stirred under a nitrogen atmosphere at room temperature for 30 min, and then diluted with ethyl acetate (20 mL). The $Al(CH_3)_3$ excess was cautiously destroyed with a 1M NaOH solution (2.0 mL) and the resulting mixture was partitioned between water and ethyl acetate. The organic layer was dried over sodium sulfate and concentrated under reduced pressure. Purification of the residue by flash chromatography with the indicated eluent afforded the expected indenol derivatives (**2a-g**).

Ethyl 1-hydroxy-6-methoxy-3-(2-methoxyphenyl)-1-methyl-1*H*-indene-2-carboxylate (3a).

The title compound was obtained starting from indenone derivative **2a** (0.59 g, 1.74 mmol) as an orange oil (0.56 g, yield 91%) using petroleum ether-ethyl acetate (8:2 v/v) as the eluting mixture. ^{1}H-NMR (400 MHz, $CDCl_3$): 0.99 (t, *J* = 6.9, 3H), 1.77 (s, 3H), 3.71 (s, 3H), 3.85 (s, 3H), 4.08 (m, 2H), 6.80 (dd, *J* = 8.3, 2.3, 1H), 6.95-7.02 (m, 3H), 7.10 (d, *J* = 2.2, 1H), 7.20 (d, *J* = 6.8, 1H), 7.36 (t, *J* = 7.1, 1H). MS(ESI): m/z 377 (M+Na$^+$).

Ethyl 1-hydroxy-6-methoxy-3-(3-methoxyphenyl)-1-methyl-1*H*-indene-2-carboxylate (3b).

The title compound was obtained starting from indenone derivative **2b** (0.16 g, 0.47 mmol) as an orange solid (0.15 g, yield 90%, mp 71-72 °C) using petroleum ether-ethyl acetate (9:1 v/v) as the eluting mixture. ^{1}H-NMR (400 MHz, $CDCl_3$): 1.06 (t, *J* = 7.1, 3H), 1.76 (s, 3H), 3.70 (s, 1H), 3.82 (s, 3H), 3.86 (s, 3H), 4.12 (m, 2H), 6.80 (dd, *J* = 8.4, 2.2, 1H), 6.91-6.97 (m, 3H), 7.07 (d, *J* = 8.4, 1H), 7.14 (d, *J* = 2.2, 1H), 7.34 (t, *J* = 7.9, 1H). MS(ESI): m/z 377 ($M+Na^+$).

Ethyl 1-hydroxy-6-methoxy-3-(4-methoxyphenyl)-1-methyl-1*H*-indene-2-carboxylate (3c).
The title compound was obtained starting from indenone derivative **2c** (0.89 g, 2.63 mmol) as an orange solid (0.62 g, yield 67%, mp 101-102 °C) using petroleum ether-ethyl acetate (9:1 v/v) as the eluting mixture. ^{1}H-NMR (200 MHz, $CDCl_3$): 1.09 (t, *J* = 7.1, 3H), 1.73 (s, 3H), 3.82 (s, 6H), 4.10 (m, 2H), 6.78 (dd, *J* = 8.3, 2.4, 1H), 6.93 (d, *J* = 8.7, 2H), 7.08 (m, 2H), 7.33 (d, *J* = 8.7, 2H). MS(ESI): m/z 377 ($M+Na^+$).

Ethyl 3-(3,4-dimethoxyphenyl)-1-hydroxy-6-methoxy-1-methyl-1*H*-indene-2-carboxylate (3d).
The title compound was obtained starting from indenone derivative **2d** (0.48 g, 1.30 mmol) as a yellow solid (0.40 g, yield 80%, mp 105-106 °C) using petroleum ether-ethyl acetate (8:2 v/v) as the eluting mixture. ^{1}H-NMR (400 MHz, $CDCl_3$): 1.11 (t, *J* = 7.1, 3H), 1.75 (s, 3H), 3.86 (s, 3H) 3.87 (s, 3H), 3.93 (s, 3H) 4.16 (m, 2H), 6.81 (dd, *J* = 8.4, 2.0, 1H), 6.93-6.98 (m, 3H), 7.12 (m, 2H). MS(ESI): m/z 407 ($M+Na^+$).

Ethyl 1-hydroxy-6-methoxy-1-methyl-3-(3,4,5-trimethoxyphenyl)-1*H*-indene-2-carboxylate (3e).
The title compound was obtained starting from indenone derivative **2e** (0.13 g, 0.326 mmol) as a yellow solid (0.12 g, yield 89%, mp 138-140 °C) using petroleum ether-ethyl acetate (8:2 v/v) as the eluting mixture. An analytical sample was obtained by recrystallization from ethyl acetate by slow evaporation. ^{1}H-NMR (400 MHz, $CDCl_3$): 1.10 (t, *J* = 7.1, 3H), 1.76 (s, 3H), 3.85 (s, 6H), 3.87

(s, 3H), 3.90 (s, 3H), 4.15 (m, 2H) 6.59 (s, 2H), 6.82 (dd, J = 8.4, 2.2, 1H), 7.13 (m, 2H). MS(ESI): m/z 437 (M+Na$^+$).

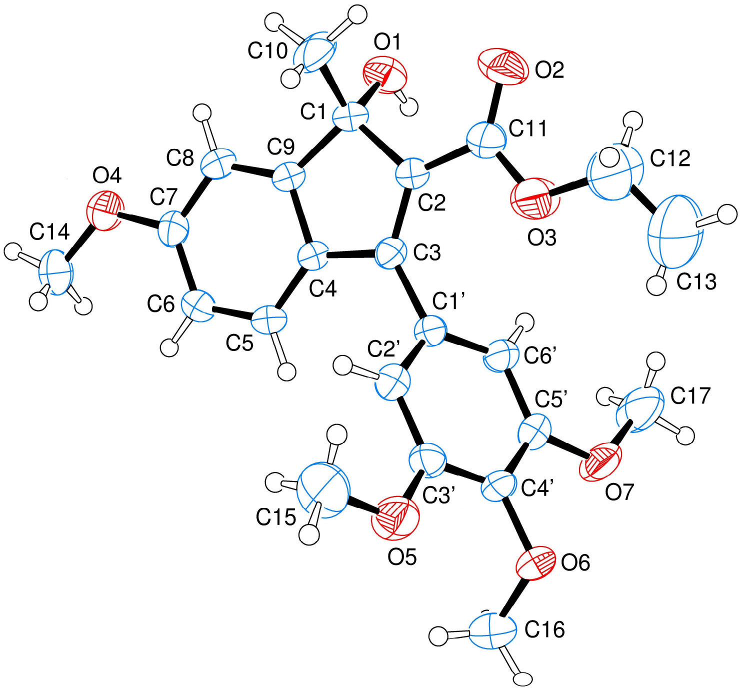

Figure S1. X-ray crystal structure of **3e**. Ellipsoids enclose 50% probability.

Ethyl 1-hydroxy-6-methoxy-1-methyl-3-(4-nitrophenyl)-1*H*-indene-2-carboxylate (3f).

The title compound was obtained starting from indenone derivative **2f** (0.29 g, 0.82 mmol) as a yellow solid (0.23 g, yield 76%, mp 159-160 °C) using petroleum ether-ethyl acetate (8:2 v/v) as the eluting mixture. An analytical sample was obtained by crystallization from ethyl acetate by slow evaporation. ^{1}H-NMR (400 MHz, $CDCl_3$): 1.05 (t, J = 7.1, 3H), 1.78 (s, 3H), 3.61 (br s, 1H), 3.87 (s, 3H), 4.12 (m, 2H), 6.82 (dd, J = 8.4, 2.4, 1H), 6.94 (d, J = 8.4, 1H), 7.16 (d, J = 2.3, 1H), 7.55 (d, J = 8.8, 2H), 8.31 (d, J = 8.8, 2H). MS(ESI): m/z 392 (M+Na$^+$).

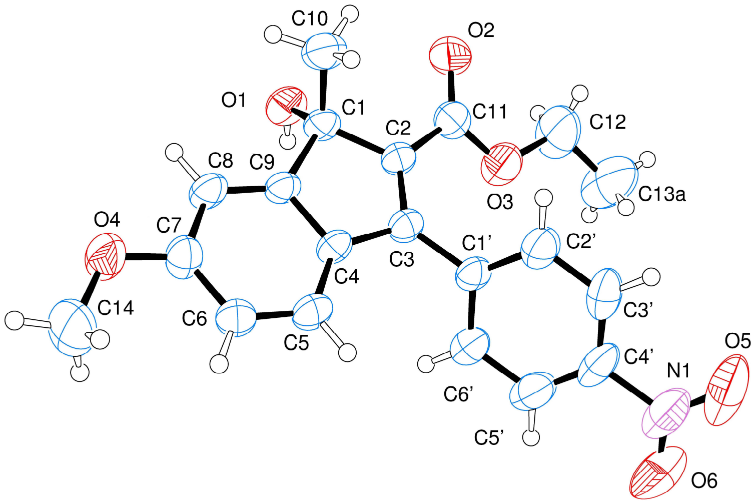

Figure S2. X-ray crystal structure of **3f**. Site occupation factor of C12a is 0.61(2). Ellipsoids enclose 50% probability.

Ethyl 3-(4-bromophenyl)-1-hydroxy-6-methoxy-1-methyl-1*H*-indene-2-carboxylate (3g).

The title compound was obtained starting from indenone derivative **2g** (0.30 g, 0.775 mmol) as a yellow solid (0.22 g, yield 70%, mp 121-122 °C) using petroleum ether-ethyl acetate (8:2 v/v) as the eluting mixture. An analytical sample was obtained by recrystallization from ethyl acetate by slow evaporation. ^{1}H-NMR (400 MHz, $CDCl_3$): 1.09 (t, *J* = 7.1, 3H), 1.75 (s, 3H), 3.65 (s, 1H), 3.85 (s, 3H), 4.13 (m, 2H) 6.80 (dd, *J* = 8.4, 2.4, 1H), 7.00 (d, *J* = 8.4, 1H), 7.13 (d, *J* = 2.3, 1H), 7.26 (d, *J* = 8.4, 2H), 7.57 (d, *J* = 8.4, 2H). MS(ESI): m/z 425, 427 (M+Na$^+$).

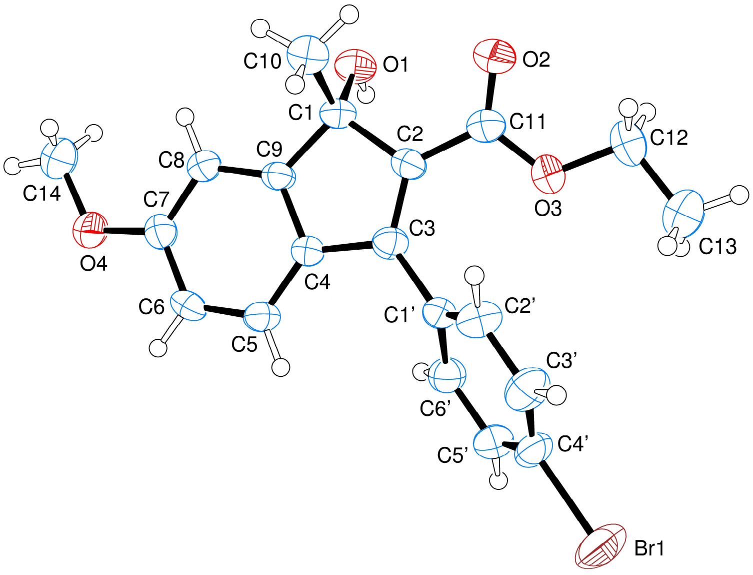

Figure S3. X-ray crystal structure of **3g**. Ellipsoids enclose 50% probability.

Ethyl 3-(furan-3-yl)-1-hydroxy-6-methoxy-1-methyl-1*H*-indene-2-carboxylate (3h).

To a solution of indenone derivative **2h** (0.045 g, 0.15 mmol) in dichloromethane (20 mL) was added a 3M solution of CH_3MgBr in diethyl ether (0.30 mL, 0.90 mmol). The reaction mixture was stirred under a nitrogen atmosphere at room temperature for 15 min. The CH_3MgBr excess was cautiously decomposed with a saturated solution of NH_4Cl and the resulting mixture was dried over sodium sulfate and concentrated under reduced pressure. Purification of the residue by flash chromatography with petroleum ether–ethyl acetate (9:1 v/v) as the eluent afforded indenol derivatives **3h** as a yellow oil (0.020 g, yield 42%). ^{1}H-NMR (400 MHz, $CDCl_3$): 1.29 (t, J = 7.1, 3H), 1.72 (s, 3H), 3.71 (s, 1H), 3.87 (s, 3H), 4.27 (m, 2H), 6.67 (s, 1H), 6.85 (dd, J = 8.4, 2.4, 1H), 7.12 (d, J = 2.3, 1H), 7.32 (d, J = 8.4, 1H), 7.51 (s, 1H), 7.78 (s, 1H). MS(ESI): m/z 337 ($M+Na^+$).

General procedure for the preparation of solutions of monomers 1a-h in Chloroform or $CDCl_3$.

A mixture of the appropriate indenol derivative (**3a-h**) in chloroform or $CDCl_3$ (20 mL/1 mmol of indenol) with a catalytic amount of *p*-toluenesulfonic acid monohydrate (PTSA) (0.2 equivalents) was heated under reflux for 1-2 h and cooled to room temperature monitoring the reaction by TLC. The reaction mixture was then washed with a saturated solution of $NaHCO_3$ and dried over sodium sulfate to afford a stock (about 0.05 M) solution of the corresponding monomer (**1a-h**).

Ethyl 6-methoxy-3-(2-methoxyphenyl)-1-methylene-1*H*-indene-2-carboxylate (1a).

This monomer was prepared from indenol **3a** (0.23 g, 0.65 mmol). ^{1}H-NMR (400 MHz, $CDCl_3$): 1.01 (t, J = 7.1, 3H), 3.73 (s, 3H), 3.86 (s, 3H), 4.07 (q, J = 7.1, 2H), 6.30 (s, 1H), 6.59 (s, 1H), 6.80 (dd, J = 8.3, 2.1, 1H), 6.96-7.04 (m, 3H), 7.25 (m, 2H), 7.36 (t, J = 7.7, 1H). MS(ESI): m/z 359 ($M+Na^+$).

Ethyl 6-methoxy-3-(3-methoxyphenyl)-1-methylene-1*H*-indene-2-carboxylate (1b). This monomer was prepared from indenol **3b** (0.23 g, 0.65 mmol). ^{1}H-NMR (400 MHz, $CDCl_3$): 1.06 (t, *J* = 7.1, 3H), 3.82 (s, 3H), 3.87 (s, 3H), 4.11 (q, *J* = 7.1, 2H), 6.33 (s, 1H), 6.59 (s, 1H), 6.82 (dd, *J* = 8.4, 2.4, 1H), 6.93-6.99 (m, 3H), 7.13 (d, *J* = 8.4, 1H), 7.25 (s, 1H), 7.34 (t, *J* = 8.2, 1H). MS(ESI): m/z 359 ($M+Na^+$).

Ethyl 6-methoxy-3-(4-methoxyphenyl)-1-methylene-1*H*-indene-2-carboxylate (1c). This monomer was prepared from indenol **3c** (0.62 g, 1.76 mmol). ^{1}H-NMR (400 MHz, $CDCl_3$): 1.12 (t, *J* = 7.1, 3H), 3.86 (s, 3H), 3.87 (s, 3H), 4.15 (q, *J* = 7.1, 2H), 6.30 (s, 1H), 6.55 (s, 1H), 6.83 (dd, *J* = 8.4, 2.3, 1H), 6.97 (d, *J* = 8.8, 2H), 7.16 (d, *J* = 8.4, 1H), 7.25 (d, *J* = 2.4, 1H), 7.37 (d, *J* = 8.8, 2H). MS(ESI): m/z 359 ($M+Na^+$).

Ethyl 3-(3,4-dimethoxyphenyl)-6-methoxy-1-methylene-1*H*-indene-2-carboxylate (1d). This monomer was prepared from indenol **3d** (0.32 g, 0.83 mmol). ^{1}H-NMR (400 MHz, $CDCl_3$): 1.12 (t, *J* = 7.1, 3H), 3.88 (s, 6H), 3.94 (s, 3H), 4.15 (q, *J* = 7.1, 2H), 6.31 (s, 1H), 6.55 (s, 1H), 6.84 (dd, *J* = 8.4, 2.3, 1H), 6.94-7.02 (m, 3H), 7.17 (d, *J* = 8.4, 1H), 7.24 (s, 1H). MS(ESI): m/z 389 ($M+Na^+$).

Ethyl 6-methoxy-1-methylene-3-(3,4,5-trimethoxyphenyl)-1*H*-indene-2-carboxylate (1e). This monomer was prepared from indenol **3e** (0.43 g, 1.04 mmol). ^{1}H-NMR (400 MHz, $CDCl_3$): 1.11 (t, *J* = 7.6, 3H), 3.85 (s, 6H), 3.88 (s, 3H), 3.91 (s, 3H), 4.15 (q, *J* = 7.1, 2H), 6.33 (s, 1H), 6.57 (s, 1H), 6.63 (s, 2H), 6.85 (dd, *J* = 8.4, 2.0, 1H), 7.19 (d, *J* = 8.4, 2H), 7.24 (s, 1H). MS(ESI): m/z 419 ($M+Na^+$).

Ethyl 6-methoxy-1-methylene-3-(4-nitrophenyl)-1*H*-indene-2-carboxylate (1f).

This monomer was prepared from indenol **3f** (0.30 g, 0.81 mmol). ^{1}H-NMR (400 MHz, $CDCl_3$): 1.07 (t, *J* = 7.1, 3H), 3.88 (s, 3H), 4.12 (q, *J* = 7.1, 2H), 6.42 (s, 1H), 6.72 (s, 1H), 6.84 (dd, *J* = 8.4, 2.2, 1H), 6.98 (d, *J* = 8.4, 1H), 7.28 (d, *J* = 2.2, 1H), 7.56 (d, *J* = 8.8, 2H), 8.31 (d, *J* = 8.8, 2H). MS(ESI, negative ions): m/z 701 ($2M-H^+$).

Ethyl 3-(4-bromophenyl)-6-methoxy-1-methylene-1*H*-indene-2-carboxylate (1g).

This monomer was prepared from indenol **3g** (0.22 g, 0.54 mmol). ^{1}H-NMR (400 MHz, $CDCl_3$): 1.09 (t, *J* = 7.1, 3H), 3.87 (s, 3H), 4.13 (q, *J* = 7.1, 2H), 6.35 (s, 1H), 6.63 (s, 1H), 6.83 (dd, *J* = 8.4, 2.3, 1H), 7.06 (d, *J* = 8.4, 1H), 7.25 (d, *J* = 2.4, 1H), 7.27 (d, *J* = 8.4, 2H), 7.57 (d, *J* = 8.4, 2H). MS(ESI): m/z 407, 409 ($M+Na^+$).

Ethyl 3-(furan-3-yl)-6-methoxy-1-methylene-1*H*-indene-2-carboxylate (1h).

This monomer was prepared from indenol **3h** (0.25 g, 0.80 mmol) and 1 equivalent of PTSA. ^{1}H-NMR (400 MHz, $CDCl_3$): 1.30 (t, *J* = 7.2, 3H), 3.88 (s, 3H), 4.28 (q, *J* = 7.1, 2H), 6.29 (s, 1H), 6.49 (s, 1H), 6.68 (s, 1H), 6.87 (dd, *J* = 8.4, 2.3, 1H), 7.24 (d, *J* = 2.2, 1H), 7.36 (d, *J* = 8.4, 1H), 7.52 (s, 1H) 7.84 (s, 1H). MS (ESI): m/z 319 ($M+Na^+$).

General procedure for the preparation of polybenzofulvene derivatives poly-1b-e,g,h by spontaneous polymerization.

A stock solution of suitable monomer (**1b-e,g,h**) in chloroform (about 0.05 M) was concentrated under reduced pressure to give a viscous oil, which was dissolved into chloroform (20 mL/mmol of monomer) and newly evaporated. The dissolution/evaporation procedure was repeated from three to nine times monitoring by TLC the progressive disappearance of the monomer in solution. The final residue was washed with the indicated solvent or dissolved into a small amount of $CHCl_3$ and precipitated in the reported bad solvent to give the expected polymer.

Poly-[Ethyl 6-methoxy-3-(3-methoxyphenyl)-1-methylene-1*H*-indene-2-carboxylate] (Poly-1b).

This polymer was obtained from a stock solution of benzofulvene monomer **1b** in chloroform. The dissolution/evaporation procedure was repeated three times and the final residue was dissolved into a small amount of $CHCl_3$ and precipitated with ethanol to give poly-**1b** as a white solid (0.12 g, yield from **3b** 55%). ^{1}H-NMR spectrum ($CDCl_3$, 300 MHz): see Figure S4. ^{13}C-NMR spectrum ($CDCl_3$, 75 MHz): see Figure S27.

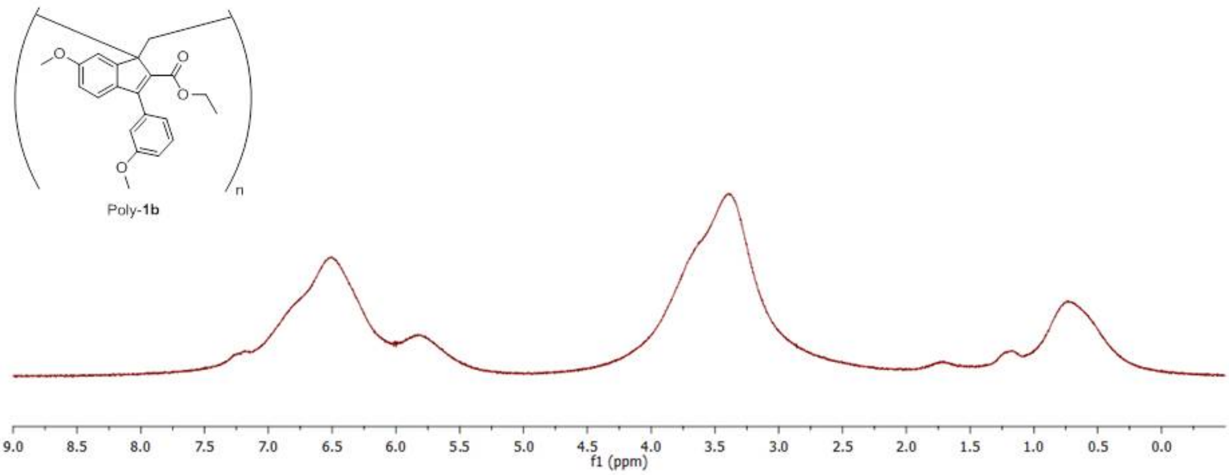

Figure S4. ^{1}H-NMR spectrum ($CDCl_3$, 300 MHz) of poly-**1b**.

Poly-[Ethyl 6-methoxy-3-(4-methoxyphenyl)-1-methylene-1*H*-indene-2-carboxylate] (Poly-1c). This polymer was obtained from a stock solution of benzofulvene monomer **1c** in chloroform. The dissolution/evaporation procedure was repeated three times and the final residue was dissolved into a small amount of $CHCl_3$ and precipitated with methanol to give poly-**1c** as a white powder (0.41 g, yield from **3c** 69%). ^{1}H-NMR spectrum ($CDCl_3$, 300 MHz): see Figure S5. ^{13}C-NMR spectrum ($CDCl_3$, 75 MHz): see Figure S28.

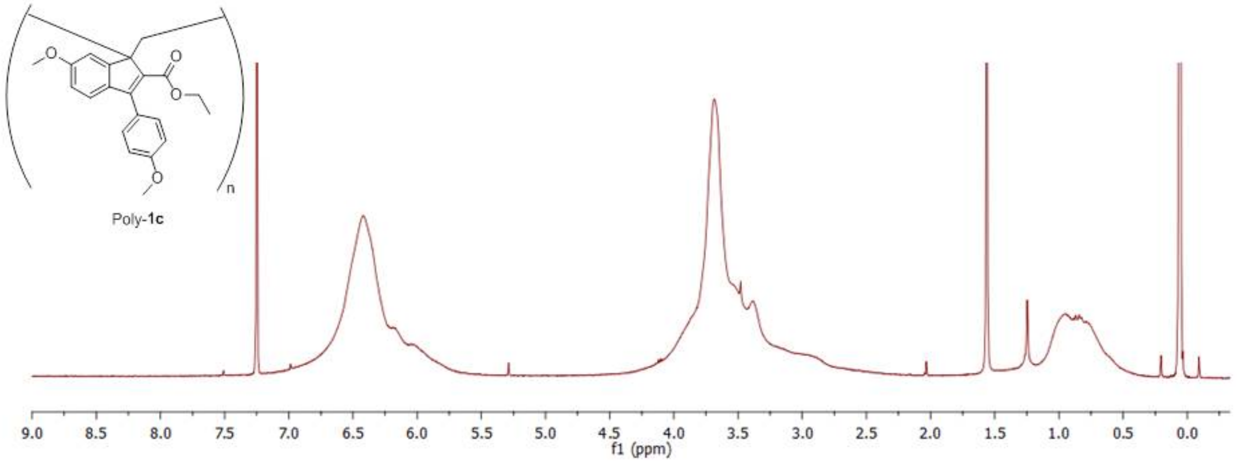

Figure S5. ^{1}H-NMR spectrum ($CDCl_3$, 400 MHz) of poly-**1c**.

Poly-[Ethyl 3-(3,4-dimethoxyphenyl)-6-methoxy-1-methylene-1*H*-indene-2-carboxylate] (Poly-1d).

This polymer was obtained from a stock solution of benzofulvene monomer **1d** in chloroform. The dissolution/evaporation procedure was repeated five times and the final residue was dissolved into a small amount of $CHCl_3$ and precipitated with ethanol to give poly-**1d** as a pale yellow cottony solid (0.21 g, yield from **3d** 69%). ^{1}H-NMR spectrum ($CDCl_3$, 300 MHz): see Figure S6. ^{13}C-NMR spectrum ($CDCl_3$, 75 MHz): see Figure S29.

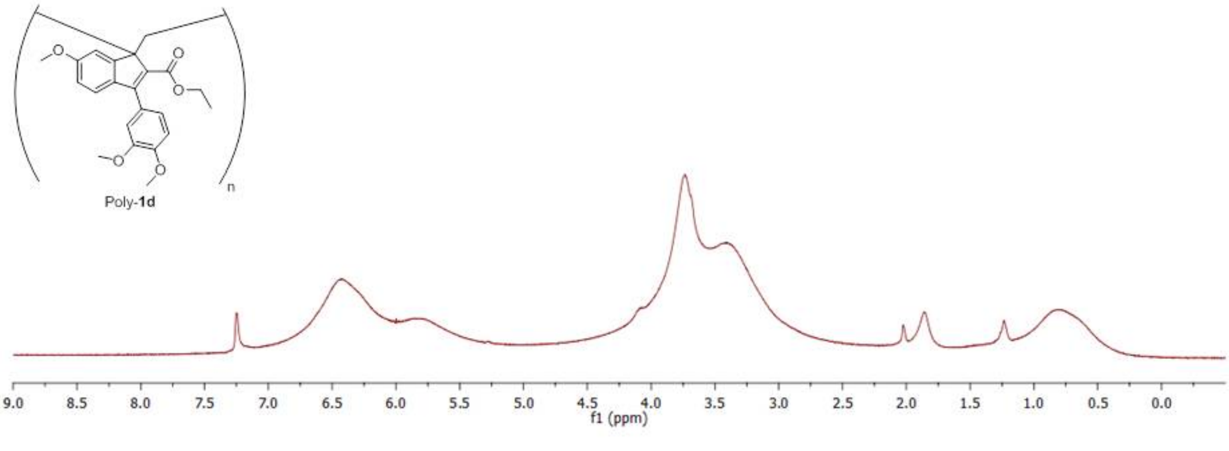

Figure S6. ^{1}H-NMR spectrum ($CDCl_3$, 300 MHz) of poly-**1d**.

Poly-[Ethyl 6-methoxy-1-methylene-3-(3,4,5-trimethoxyphenyl)-1*H*-indene-2-carboxylate] (Poly-1e).

This polymer was obtained from a stock solution of benzofulvene monomer **1e** in chloroform. The dissolution/evaporation procedure was repeated five times and the final residue was dissolved into a small amount of $CHCl_3$ and precipitated with n-hexane to give poly-**1e** as a white cottony solid (0.30 g, yield from **3e** 72%). ^{1}H-NMR spectrum ($CDCl_3$, 300 MHz): see Figure S7. ^{13}C-NMR spectrum ($CDCl_3$, 75 MHz): see Figure S30.

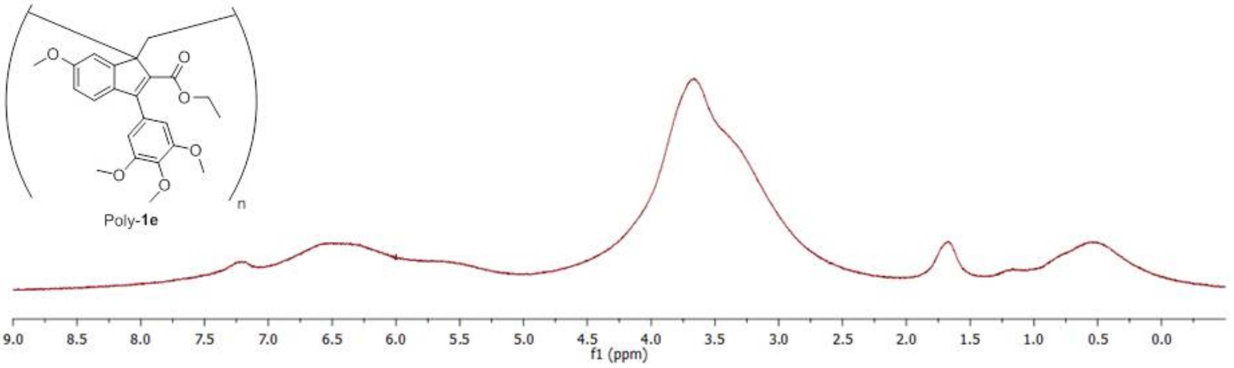

Figure S7. ^{1}H-NMR spectrum ($CDCl_3$, 300 MHz) of poly-**1e**.

Poly-[Ethyl 3-(4-bromophenyl)-6-methoxy-1-methylene-1*H*-indene-2-carboxylate] (Poly-1g).

This polymer was obtained from a stock solution of benzofulvene monomer **1g** in chloroform. The dissolution/evaporation procedure was repeated three times and the final residue was purified by washing with n-hexane to give poly-**1g** as a white solid (0.13 g, yield from **3g** 63%). ^{1}H-NMR spectrum ($CDCl_3$, 300 MHz): see Figure S8. ^{13}C-NMR spectrum ($CDCl_3$, 75 MHz): see Figure S32.

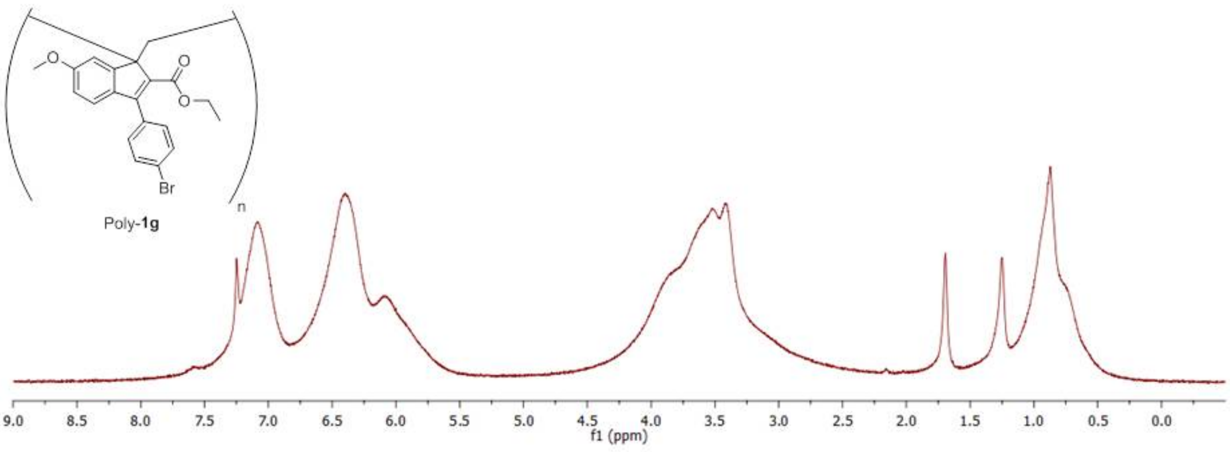

Figure S8. ^{1}H-NMR spectrum ($CDCl_3$, 300 MHz) of poly-**1g**.

Poly-[Ethyl 3-(furan-3-yl)-6-methoxy-1-methylene-1*H*-indene-2-carboxylate] (Poly-1h).

This polymer was obtained from a stock solution of benzofulvene monomer **1h** in chloroform. The dissolution/evaporation procedure was repeated nine times and the final residue was dissolved into a small amount of $CHCl_3$ and precipitated with n-hexane to give poly-**1h** as a beige powder (0.13 g, yield from **3h** 55%). ^{1}H-NMR spectrum ($CDCl_3$, 400 MHz): see Figure S9. ^{13}C-NMR spectrum ($CDCl_3$, 75 MHz): see Figure S33

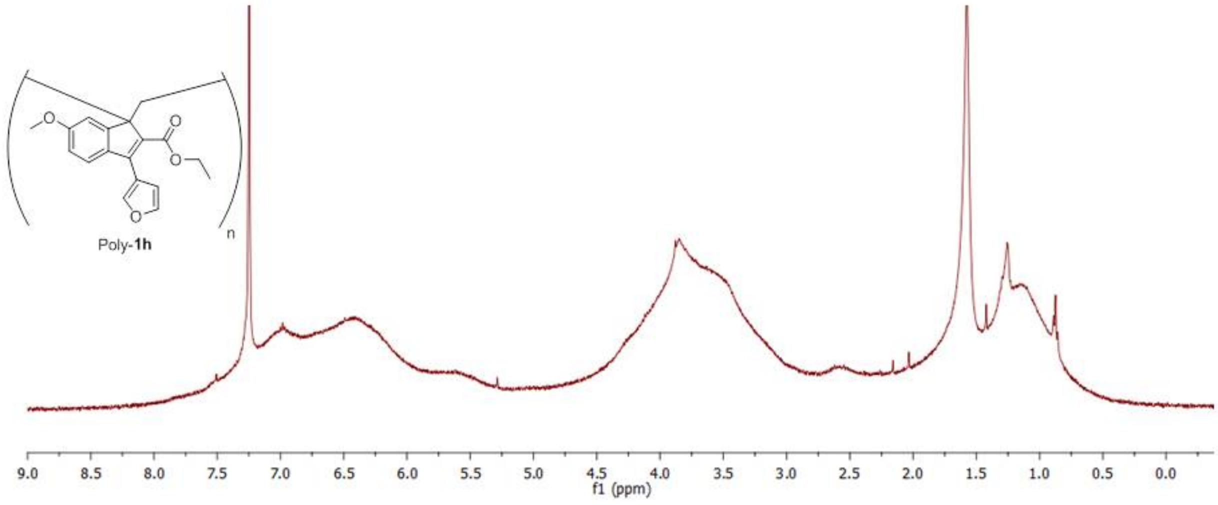

Figure S9. ^{1}H-NMR spectrum ($CDCl_3$, 400 MHz) of poly-**1h**.

Poly-[Ethyl 6-methoxy-3-(2-methoxyphenyl)-1-methylene-1*H*-indene-2-carboxylate] (Poly-1a). The stock solution of benzofulvene monomer **1a** in chloroform (about 0.05 M) was concentrated under reduced pressure to give a viscous oil, which was dissolved into chloroform (20 mL/mmol of monomer) and newly evaporated. The dissolution/evaporation procedure was repeated several times without the complete disappearance of the monomer (the progress of the polymerization process was monitored by TLC). The final residue was dissolved into a small amount of $CHCl_3$ and precipitated with ethanol to give poly-**1a** as a white solid (0.070 g, yield from **3a** 32%). ^{1}H-NMR spectrum ($CDCl_3$, 300 MHz): see Figure S10. ^{13}C-NMR spectrum ($CDCl_3$, 75 MHz): see Figure S26.

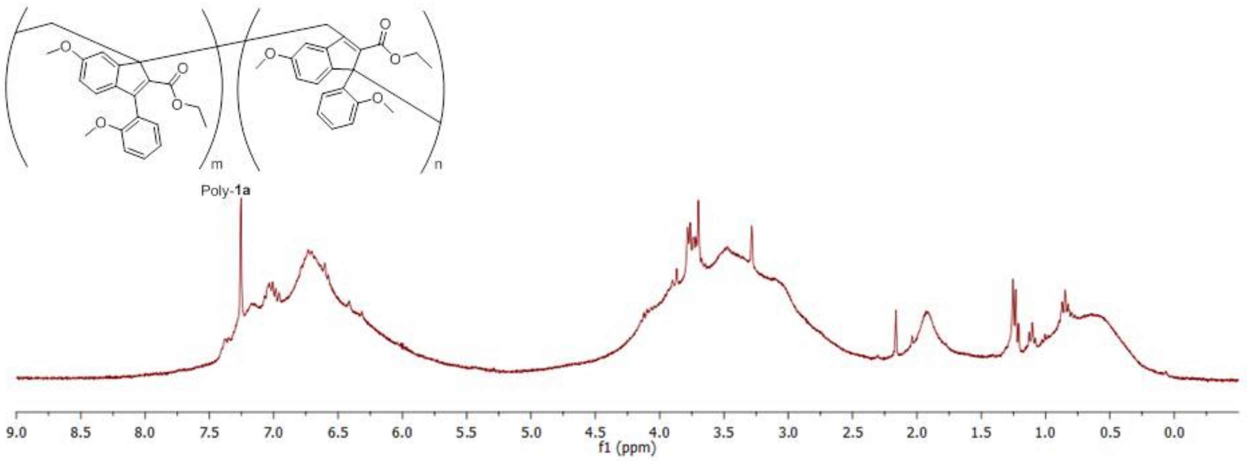

Figure S10. ^{1}H-NMR spectrum ($CDCl_3$, 300 MHz) of poly-**1a**.

Poly-[Ethyl 6-methoxy-1-methylene-3-(4-nitrophenyl)-1*H*-indene-2-carboxylate] (Poly-1f). The stock solution of benzofulvene monomer **1f** in chloroform (about 0.05 M) was concentrated under reduced pressure to give a viscous brown oil. The oil was suspended into chloroform (10.0 mL/mmol of monomer) to obtain a yellow precipitate. The precipitate was collected by filtration, dissolved in a small amount of dichloromethane and newly precipitated in ethanol to obtain poly-**1e**

as a yellow powder (0.22 g, yield from **3f** 77%).^{1}H-NMR spectrum ($CDCl_3$, 300 MHz): see Figure S11. ^{13}C-NMR spectrum (CD_2Cl_2, 75 MHz): see Figure S31.

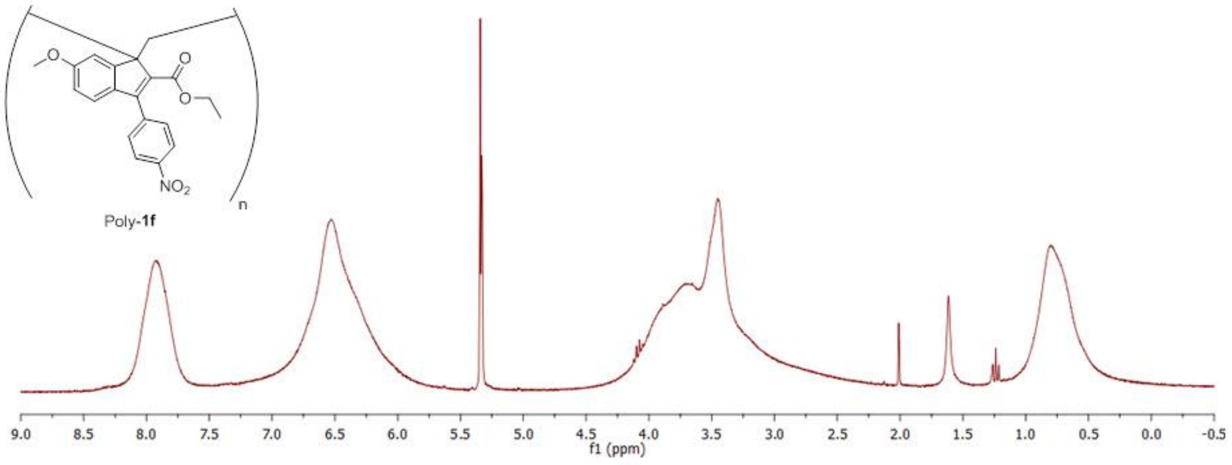

Figure S11. ^{1}H-NMR spectrum (CD_2Cl_2, 300 MHz) of poly-**1f**.

General procedure for the synthesis of compounds 5a-e,h.

A mixture of the appropriate β-ketoester (**4a-e,h**) (1 equivalent) in DMF (15 mL) with K_2CO_3 (3 equivalents), NaI (1.5 equivalents), and 3-methoxybenzylchloride (1 equivalent) was stirred at room temperature for 4-16 h. The reaction mixture was then diluted with a saturated NH_4Cl solution and extracted with ethyl acetate. The organic layer was dried over sodium sulfate and evaporated under reduced pressure. Purification of the residue by flash chromatography with petroleum ether-ethyl acetate (9:1) as the eluent gave the expected compound (**5a-e,h**).

Ethyl 2-(3-methoxybenzyl)-3-(2-methoxyphenyl)-3-oxopropanoate (5a).

Compound **5a** (0.83 g, yield 79%) was obtained as a colorless oil starting from ethyl (2-methoxybenzoyl)acetate (**4a**, 0.70 g, 3.15 mmol) and using $NaHCO_3$ as base instead of K_2CO_3. Furthermore, the reaction mixture was stirred at about 100° C for 16 h. ^{1}H-NMR (200 MHz, $CDCl_3$): 1.11 (t, J = 7.1, 3H), 3.11-3.34 (m, 2H), 3.74 (s, 3H), 3.84 (s, 3H), 4.08 (q, J = 7.1, 2H),

4.63 (t, J = 7.3, 1H), 6.68-6.81 (m, 3H) 6.89-7.00 (m, 2H), 7.14 (t, J = 7.7,1H), 7.45 (t, J = 7.7, 1H), 7.66 (d, J = 7.2, 1H). MS(ESI): m/z 365 ($M+Na^+$).

Ethyl 2-(3-methoxybenzyl)-3-(3-methoxyphenyl)-3-oxopropanoate (5b).

Compound **5b** (0.26 g, yield 86%) was obtained as a colorless oil starting from ethyl (3-methoxybenzoyl)acetate (**4b**, 0.17 mL, 0.88 mmol) after 4 h at room temperature. ^{1}H-NMR (400 MHz, $CDCl_3$): 1.12 (t, J = 7.1, 3H), 3.29 (d, J = 6.8, 2H), 3.75 (s, 3H), 3.82 (s, 3H), 4.11 (m, 2H), 4.58 (t, J = 7.3, 1H), 6.72 (dd, J = 8.2, 2.4, 1H) 6.76 (s, 1H), 6.80 (d, J = 7.6, 1H), 7.08 (dd, J = 8.2, 2.6, 1H), 7.16 (t, J = 7.8, 1H) 7.33 (t, J = 7.9, 1H), 7.46 (s, 1H), 7.53 (d, J = 7.7, 1H). MS(ESI): m/z 365 ($M+Na^+$).

Ethyl 2-(3-methoxybenzyl)-3-(4-methoxyphenyl)-3-oxopropanoate (5c).

Compound **5c** (5.0 g, yield 89%) was obtained as a colorless oil starting from ethyl (4-methoxybenzoyl)acetate (**4c**, 3.45 mL, 16.4 mmol) after 4 h at room temperature and was purified using petroleum ether-ethyl acetate (8:2) as the eluent. ^{1}H-NMR (400 MHz, $CDCl_3$): 1.12 (t, J = 7.1, 3H), 3.23-3.33 (m, 2H), 3.75 (s, 3H), 3.85 (s, 3H), 4.06-4.15 (m, 2H), 4.55 (t, J = 7.3, 1H), 6.70-6.81 (m, 3H), 6.90 (d, J = 8.9, 2H), 7.15 (t, J = 7.8, 1H), 7.94 (d, J = 8.9, 2H). MS(ESI): m/z 365 ($M+Na^+$).

Ethyl 3-(3,4-dimethoxyphenyl)-2-(3-methoxybenzyl)-3-oxopropanoate (5d).

Compound **5d** (0.68 g, yield 92%) was obtained as a colorless oil starting from ethyl (3,4-methoxybenzoyl)acetate (**4d**, 0.50 g, 1.98 mmol) after 15 h at room temperature. ^{1}H-NMR (400 MHz, $CDCl_3$): 1.12 (t, J = 7.1, 3H), 3.28 (m, 2H), 3.74 (s, 3H), 3.89 (s, 3H), 3.91 (s, 3H), 4.09 (m, 2H), 4.56 (t, J = 7.3, 1H), 6.71 (d, J = 8.2, 1H), 6.75 (s, 1H), 6.79 (d, J = 7.6, 1H), 6.85 (d, J = 8.4, 1H), 7.15 (t, J = 7.9, 1H), 7.50 (s, 1H), 7.60 (dd, J = 8.4, 1.5, 1H). MS (ESI): m/z 395 ($M+Na^+$).

Ethyl 2-(3-methoxybenzyl)-3-oxo-3-(3,4,5-trimethoxyphenyl)propanoate (5e).

Compound **5e** (0.90 g, yield 90%) was obtained as a colorless oil starting from ethyl (3,4,5-methoxybenzoyl)acetate (**4e**, 0.70 g, 2.48 mmol) after 15 h at room temperature. ^{1}H-NMR (200 MHz, $CDCl_3$): 1.13 (t, J = 7.2, 3H), 3.27 (m, 2H), 3.75 (s, 3H), 3.85 (s, 6H), 3.88 (s, 3H), 4.11 (q, J = 7.1, 2H), 4.52 (t, J = 7.3, 1H), 6.70-6.80 (m, 3H), 7.11-7.24 (m, 3H). MS (ESI): m/z 425 ($M+Na^+$).

Ethyl 3-(furan-3-yl)-2-(3-methoxybenzyl)-3-oxopropanoate (5h) [2].

Compound **5h** (0.76 g, yield 92%) was obtained as a colorless oil starting from ethyl 3-(furan-3-yl)-3-oxopropanoate (**4h**, 0.50 g, 2.74 mmol) after 16 h at room temperature. ^{1}H-NMR (200 MHz, $CDCl_3$): 1.13 (t, J = 7.0, 3H), 3.24 (d, J = 7.4, 2H), 3.71 (s, 3H), 4.04-4.20 (m, 3H) 6.67-6.74 (m, 4H), 7.13 (m, 1H), 7.39 (s, 1H), 8.04 (s, 1H). MS (ESI): m/z 325 ($M+Na^+$).

General procedure for the preparation of compounds 6a-e,h.

The appropriate ketone derivative (**5a-e,h**) was mixed with polyphosphoric acid (10 parts with respect to the ketone derivative) by mechanical stirring at room temperature for 1h. The reaction mixture was then diluted with water and extracted with ethyl acetate. The organic layer was dried over sodium sulfate and concentrated under reduced pressure. Purification of the residue by flash chromatography with the indicated eluent gave the expected indene derivative (**6a-e,h**).

Ethyl 6-methoxy-3-(2-methoxyphenyl)-1*H*-indene-2-carboxylate (6a).

Compound **6a** (0.58 g, yield 75%) was obtained starting from compound **5a** (0.82 g, 2.39 mmol) as a pale yellow oil using petroleum ether-ethyl acetate (9:1 v/v) as the eluent. ^{1}H-NMR (400 MHz, $CDCl_3$): 1.08 (t, J = 7.1, 3H), 3.70 (s, 3H), 3.83 (s, 5H), 4.09 (q, J = 7.1, 2H), 6.83 (dd, J = 8.4, 1.9, 1H), 6.98-7.09 (m, 4H), 7.23 (d, J = 7.4, 1H), 7.37 (t, J = 8.1, 1H). MS(ESI): m/z 347 ($M+Na^+$).

9-Methoxyindeno[2,1-c]chromen-6(7*H*)-one (7).

Compound **7** (0.050 g, yield 8%) was obtained as a secondary product from the reaction described above for the synthesis of compound **6a**. An analytical sample was obtained by recrystallization from ethyl acetate by slow evaporation (mp 185-186 °C). ^{1}H-NMR (400 MHz, $CDCl_3$): 3.91 (s, 5H), 7.06 (dd, J = 8.6, 2.1, 1H), 7.21 (s, 1H), 7.39 (t, J = 7.0, 1H), 7.47 (d, J = 7.8, 1H), 7.57 (t, J = 7.7, 1H), 8.14 (d, J = 8.6, 1H), 8.29 (d, J = 7.8, 1H). MS(ESI): 287 ($M+Na^+$).

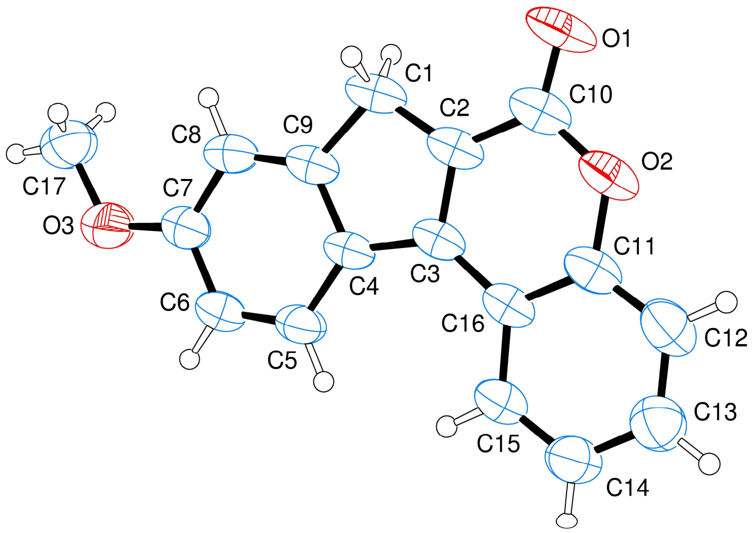

Figure S12. X-ray crystal structure of **7**. Ellipsoids enclose 50% probability.

Ethyl 6-methoxy-3-(3-methoxyphenyl)-1*H*-indene-2-carboxylate (6b).

Compound **6b** (0.13 g, yield 27%) was obtained starting from compound **5b** (0.50 g, 1.46 mmol) as a pale yellow oil using petroleum ether-ethyl acetate (9:1 v/v) as the eluent. ^{1}H-NMR (400 MHz, $CDCl_3$): 1.14 (t, J = 7.1, 3H), 3.81 (s, 2H), 3.83 (s, 3H), 3.84 (s, 3H), 4.13 (q, J = 7.1, 2H), 6.85 (d, J = 8.3, 1H), 6.94-7.00 (m, 3H), 7.10 (s, 1H), 7.20 (d, J = 8.5, 1H), 7.36 (t, J = 8.2, 1H). MS(ESI): m/z 347 ($M+Na^+$).

Ethyl 6-methoxy-3-(4-methoxyphenyl)-1*H*-indene-2-carboxylate (6c).

Compound **6c** (0.14 g, yield 51%) was obtained starting from compound **5c** (0.29 g, 0.85 mmol) as white solid using petroleum ether-ethyl acetate (9:1 v/v) as the eluent. An analytical sample was

obtained by recrystallization from *n*-hexane (mp 72-73 °C). ^{1}H-NMR (400 MHz, $CDCl_3$): 1.18 (t, *J* = 7.0, 3H), 3.80 (s, 2H), 3.84 (s, 3H), 3.86 (s, 3H), 4.14 (q, *J* = 7.0, 2H), 6.85 (dd, *J* = 8.4, 2.1, 1H), 6.98 (d, *J* = 8.6, 2H), 7.09 (d, *J* = 2.0, 1H), 7.21 (d, *J* = 8.5, 1H), 7.38 (d, *J* = 8.6, 2H). MS(ESI): m/z 347 ($M+Na^+$).

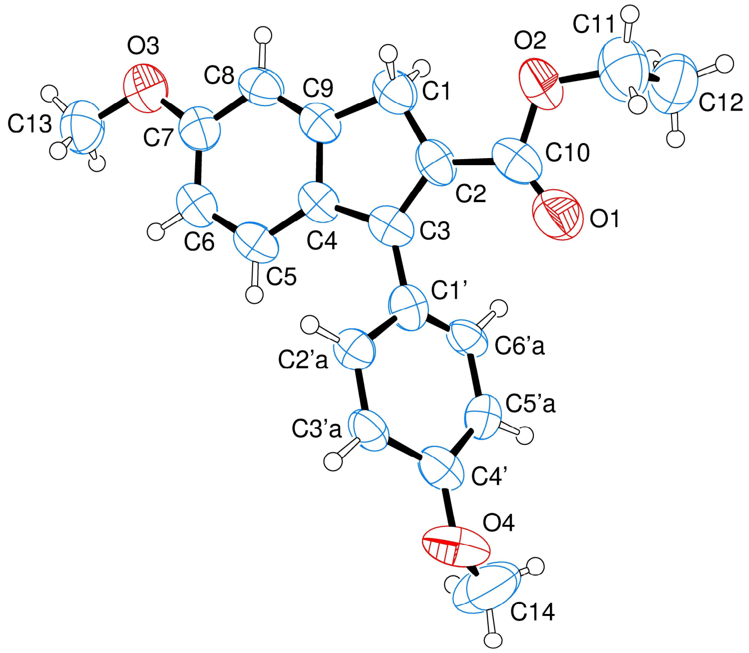

Figure S13. X-ray crystal structure of **6c**. Site occupation factor of C2', C3', C5' and C6' is 0.51(1). Ellipsoids enclose 50% probability.

Ethyl 3-(3,4-dimethoxyphenyl)-6-methoxy-1*H*-indene-2-carboxylate (6d).

The title compound **6d** (0.15 g, yield 52%) was obtained starting from compound **5d** (0.30 g, 0.81 mmol) as a white solid using petroleum ether-ethyl acetate (9:1 v/v) as the eluent. An analytical sample was obtained by recrystallization from diethyl ether by slow evaporation (mp 106-107 °C). ^{1}H-NMR (600 MHz, $CDCl_3$): 1.17 (t, *J* = 7.1, 3H), 3.80 (s, 2H), 3.85 (s, 3H), 3.87 (s, 3H), 3.92 (s, 3H), 4.14 (q, *J* = 7.1, 2H), 6.86 (dd, *J* = 8.5, 2.3, 1H), 6.95 (d, *J* = 8.2, 1H), 6.97 (d, *J* = 1.7, 1H), 7.01 (dd, *J* = 8.1, 1.7, 1H), 7.09 (d, *J* = 1.8, 1H), 7.23 (d, *J* = 8.5, 1H). MS (ESI): m/z 377 ($M+Na^+$).

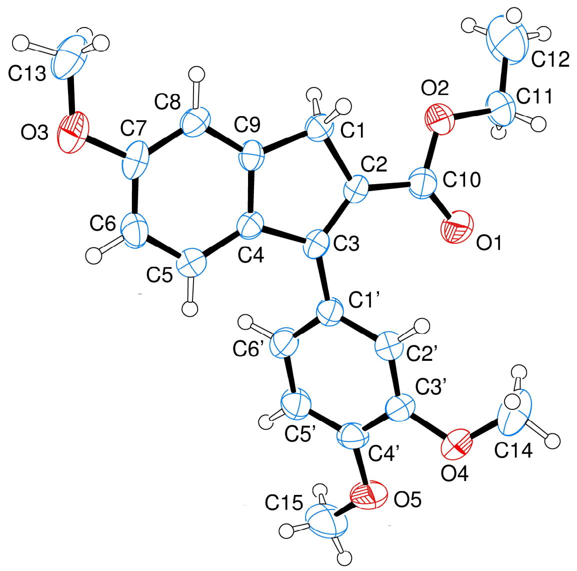

Figure S14. X-ray crystal structure of **6d**. Ellipsoids enclose 50% probability.

Ethyl 6-methoxy-3-(3,4,5-trimethoxyphenyl)-1*H*-indene-2-carboxylate (6e).

Compound **6e** (0.11 g, yield 23%) was obtained starting from compound **5e** (0.50 g, 1.24 mmol) as a white solid using petroleum ether-ethyl acetate (95:5 v/v) as the eluent. An analytical sample was obtained by recrystallization from ethyl acetate by slow evaporation (mp. 136-137 °C). ^{1}H-NMR (600 MHz, $CDCl_3$): 1.16 (t, J = 7.1, 3H), 3.81 (s, 2H), 3.85 (s, 9H), 3.91 (s, 3H), 4.13 (q, J = 7.1, 2H), 6.63 (s, 2H), 6.87 (dd, J = 8.5, 2.2, 1H), 7.11 (d, J = 2.1, 1H), 7.23 (d, J = 8.5, 1H). MS (ESI): m/z 407 (M+Na$^+$).

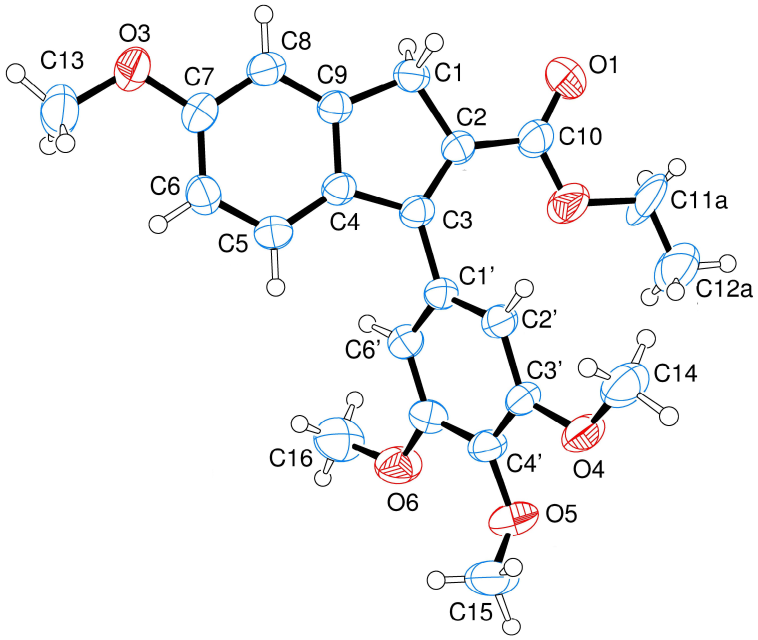

Figure S15. X-ray crystal structure of **6e**. Site occupation factor of C11A and C12A is 0.78(1). Ellipsoids enclose 50% probability.

Ethyl 3-(furan-3-yl)-6-methoxy-1H-indene-2-carboxylate (6h).

Compound **6h** (0.27 g, yield 92%) was obtained starting from compound **5h** (0.31 g, 1.03 mmol) as a white solid using petroleum ether-ethyl acetate (9:1 v/v) as the eluent. An analytical sample was obtained by recrystallization from ethyl acetate by slow evaporation (mp 106-108 °C). ^{1}H-NMR (600 MHz, $CDCl_3$): 1.29 (t, J = 7.1, 3H), 3.79 (s, 2H), 3.86 (s, 3H), 4.23 (q, J = 7.1, 2H), 6.75 (s, 1H), 6.89 (dd, J = 8.5, 2.3, 1H), 7.08 (d, J = 1.6, 1H), 7.44 (d, J = 8.5, 1H), 7.52 (s, 1H), 7.84 (s, 1H). MS (ESI): m/z 307 ($M+Na^+$).

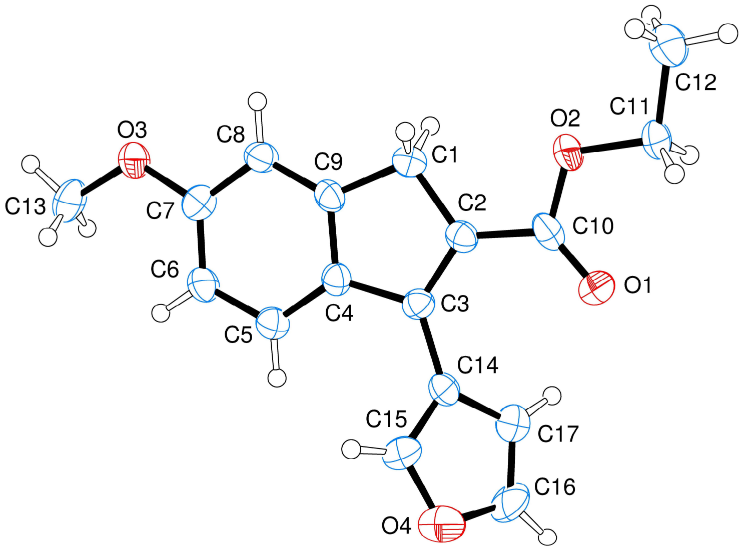

Figure S16. X-ray crystal structure of **6h**. Ellipsoids enclose 50% probability.

Ethyl 5-methoxy-1-(4-nitrophenyl)-2,3-dihydro-1*H*-indene-2-carboxylate (6f).

A mixture of ethyl (4-nitrobenzoyl)acetate (**4f**, 0.50 g, 2.11 mmol) in DMF (15 mL) with K_2CO_3 (0.88 g, 6.37 mmol), NaI (0.48 g, 3.20 mmol), and 3-methoxybenzylchloride (0.33 g, 2.11 mmol) was stirred at room temperature for 2 h. The reaction mixture was then diluted with a saturated NH_4Cl solution and extracted with ethyl acetate. The organic layer was dried over sodium sulfate and evaporated under reduced pressure. The residue was purified by flash chromatography with petroleum ether–ethyl acetate (9:1 v/v) as the eluent to obtain 0.50 g of a mixture (8:2) of **5f** and **8** as a pale yellow oil. The mixture was mixed with polyphosphoric acid (5.0 g) by mechanical stirring at room temperature for 30 min, then diluted with water and extracted with ethyl acetate. The organic layer was dried over sodium sulfate and concentrated under reduced pressure. Purification of the residue by flash chromatography with petroleum ether-ethyl acetate (8:2 v/v) as the eluent gave the expected indene derivative **6f** as pale yellow solid (0.32 g, yield 44%). An analytical sample was obtained by recrystallization from ethyl acetate by slow evaporation (mp 144-145 °C). ^{1}H-NMR (400 MHz, $CDCl_3$): 1.14 (t, J = 7.1, 3H), 3.85 (m, 2H), 3.86 (m, 3H), 4.13 (q, J = 7.1, 2H), 6.86 (dd, J = 8.5, 2.3, 1H), 7.05 (d, J = 8.5, 1H), 7.12 (s, 1H), 7.57 (d, J = 8.7, 2H), 8.31 (d, J = 8.7, 2H). MS(ESI): m/z 362 ($M+Na^+$).

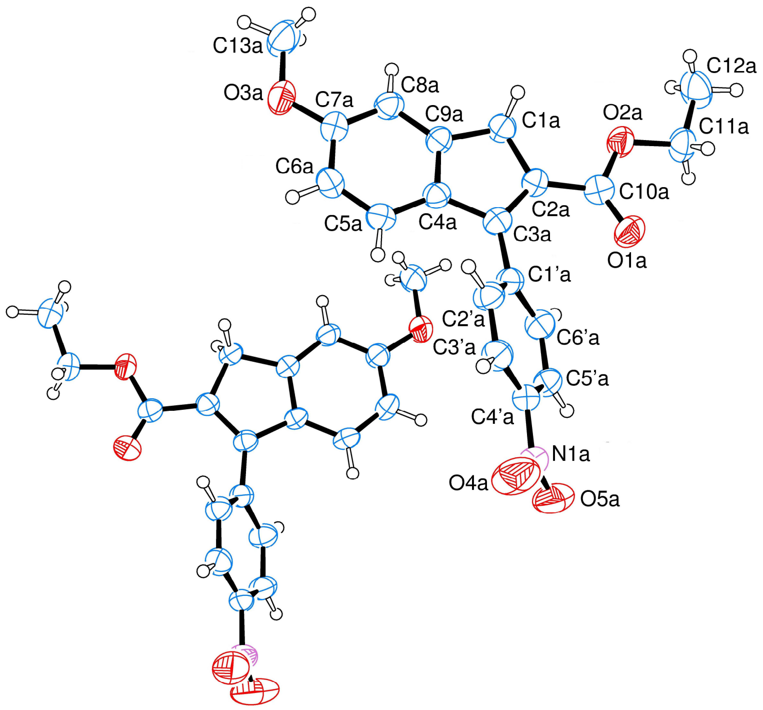

Figure S17. X-ray crystal structure of **6f**. Ellipsoids enclose 50% probability.

Ethyl 2,7-dimethoxy-4b-(4-nitrophenyl)-4b,9,9a,10-tetrahydroindeno[1,2-a]indene-9a-carboxylate (9).

Compound **9** (0.030 g, yield 6%) was obtained as a secondary product of the synthesis of compound **6f**. An analytical sample was obtained by recrystallization from dichloromethane by slow evaporation (mp 170-171 °C). ^{1}H-NMR (400 MHz, $CDCl_3$): 0.85 (t, J = 7.1, 3H), 2.93 (d, J = 16.4, 2H), 3.58 (q, J = 7.1, 2H), 3.79 (m, 8H), 6.78 (m, 4H), 6.97 (d, J = 8.3, 2H), 7.08 (d, J = 8.6, 2H), 8.03 (d, J = 8.5, 2H). MS(ESI): 482 ($M+Na^+$).

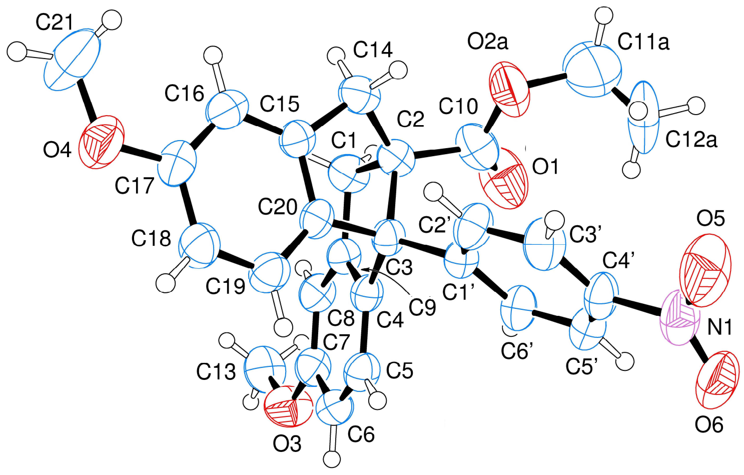

Figure S18. X-ray crystal structure of **9**. Site occupation factor of O2A, C11A and C12A is 0.70(1). Ellipsoids enclose 50% probability.

General procedure for the preparation of indanone derivatives 2a-f,h.

A mixture of appropriate indene derivative (**6a-f,h**, 1 equivalent) and SeO_2 (10 equivalents) in 1,4-dioxane (about 4 mL/mmol) was heated at reflux for 18 h. After cooling to room temperature, a saturated solution of $NaHCO_3$ was added dropwise and the resulting mixture was extracted with diethyl ether. The organic layer was dried over sodium sulfate and evaporated under reduced pressure. The residue was purified by flash chromatography with the indicated solvents as eluent to obtain the expected indenone derivative (**2a-f,h**).

Ethyl 6-methoxy-3-(2-methoxyphenyl)-1-oxo-1*H*-indene-2-carboxylate (2a).

Compound **2a** (1.1 g, yield 66%) was obtained starting from compound **6a** (1.6 g, 4.93 mmol) as a red solid using petroleum ether-ethyl acetate (8:2 v/v) as the eluent. An analytical sample was obtained by recrystallization from dichloromethane by slow evaporation (mp 135-137 °C). ^{1}H-NMR (400 MHz, $CDCl_3$): 1.10 (t, J = 7.1, 3H), 3.77 (s, 3H), 3.84 (s, 3H), 4.13 (q, J = 7.1, 2H), 6.79 (dd, J = 8.1, 2.4, 1H), 6.95-7.00 (m, 2H), 7.06 (t, J = 7.5, 1H), 7.16 (d, J = 2.4, 1H) 7.35 (dd, J = 7.6, 1.5, 1H) 7.44 (t, J = 7.1, 1H). MS(ESI): m/z 361 (M+Na$^+$).

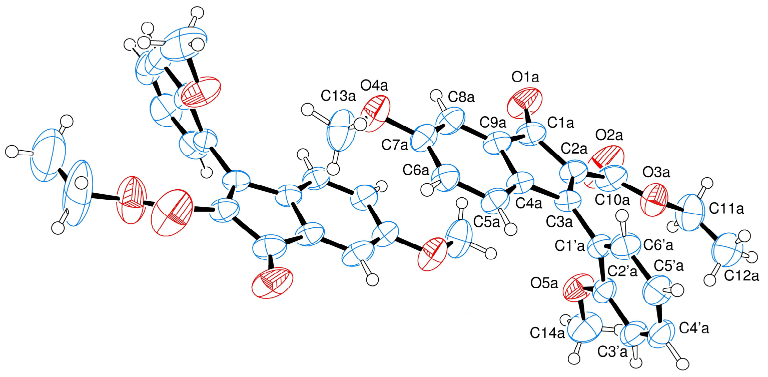

Figure S19. X-ray crystal structure of **2a**. Ellipsoids enclose 50% probability.

Ethyl 6-methoxy-3-(3-methoxyphenyl)-1-oxo-1*H*-indene-2-carboxylate (2b).

Compound **2b** (0.16 g, yield 61%) was obtained starting from compound **6b** (0.25 g, 0.77 mmol) as a red solid using petroleum ether-ethyl acetate (8:2 v/v) as the eluent. An analytical sample was obtained by recrystallization from dichloromethane by slow evaporation (mp 93-94 °C). ^{1}H-NMR (200 MHz, $CDCl_3$): 1.15 (t, J = 7.0, 3H), 3.83 (s, 3H), 3.84 (s, 3H), 4.17 (q, J = 7.2, 2H), 6.80 (dd, J = 1.8, 8.6, 1H), 7.01-7.16 (m, 5H), 7.40 (t, J = 8.3, 1H). MS(ESI): m/z 361 ($M+Na^+$).

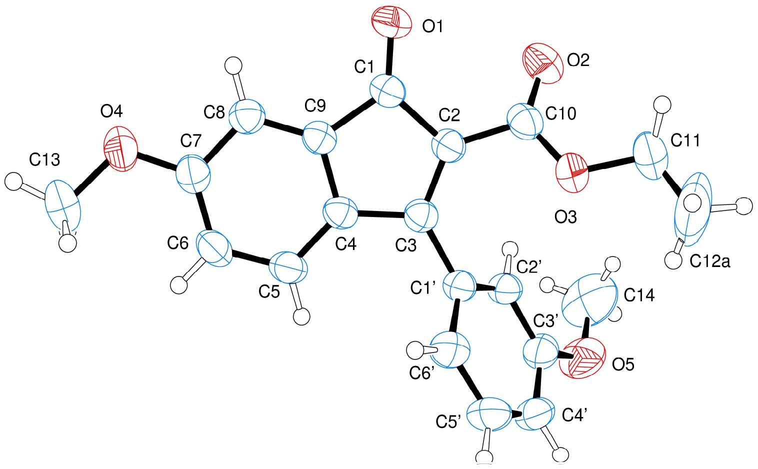

Figure S20. X-ray crystal structure of **2b**. Site occupation factor of C12a is 0.69(1). Ellipsoids enclose 50% probability.

Ethyl 6-methoxy-3-(4-methoxyphenyl)-1-oxo-1*H*-indene-2-carboxylate (2c).

Compound **2c** (0.89 g, yield 60%) was obtained starting from compound **6c** (1.42 g, 4.38 mmol) as a red solid using petroleum ether-ethyl acetate (8:2 v/v) as the eluent. An analytical sample was obtained by crystallization from diethyl ether by slow evaporation (mp 104-111 °C). ^{1}H-NMR (200 MHz, $CDCl_3$): 1.19 (t, *J* = 7.3, 3H), 3.83 (s, 3H), 3.86 (s, 3H), 4.19 (q, *J* = 7.1, 2H), 6.78 (dd, *J* = 8.6, 1.8, 1H), 6.98 (d, *J* = 8.5, 2H), 7.12 (m, 2H), 7.51 (d, *J* = 8.7, 2H). MS(ESI): m/z 361 (M+Na$^+$).

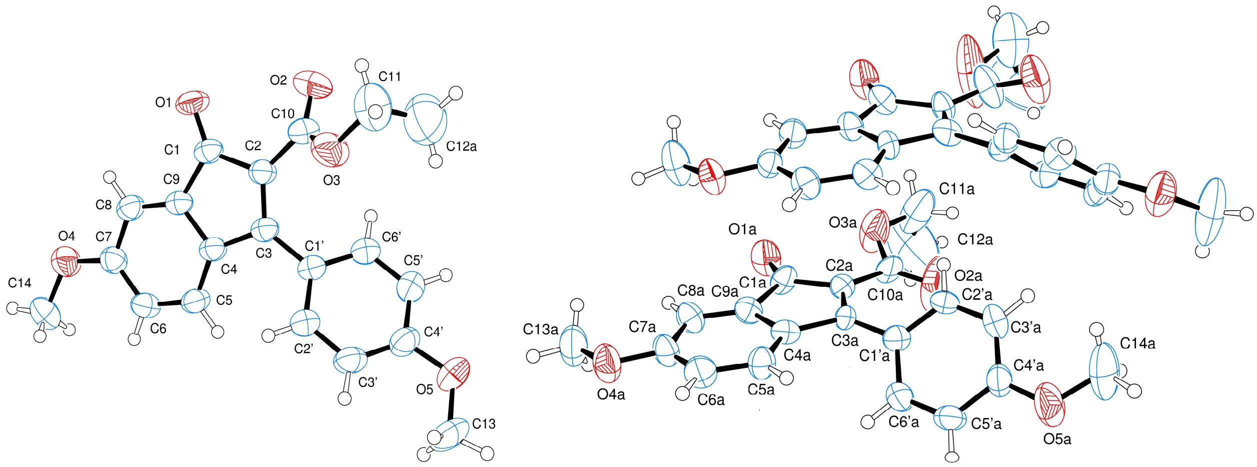

Figure S21. X-ray crystal structure of **2c**. Right: polymorph A, site occupation factor of C2', C3', C5' and C6' is 0.88(1) in both molecules of the asymmetric unit. Left: polymorph B, site occupation factor of C12a is 0.77(1). Ellipsoids enclose 50% probability.

Ethyl 3-(3,4-dimethoxyphenyl)-6-methoxy-1-oxo-1*H*-indene-2-carboxylate (2d).

Compound **2d** (0.23 g, yield 82%) was obtained starting from compound **6d** (0.27 g, 0.76 mmol) as a red solid using petroleum ether-ethyl acetate (9:1 v/v) as the eluent. An analytical sample was obtained by recrystallization from ethyl acetate by slow evaporation (mp 107-108 °C). ^{1}H-NMR (200 MHz, $CDCl_3$): 1.21 (t, *J* = 7.1, 3H), 3.85 (s, 3H), 3.89 (s, 3H), 3.95 (s, 3H), 4.21 (q, *J* = 7.1, 2H), 6.83 (dd, *J* = 8.1, 2.3, 1H), 6.99 (d, *J* = 8.1, 1H), 7.09 (d, *J* = 2.0, 1H), 7.19 (m, 3H). MS (ESI): m/z 391 (M+Na$^+$).

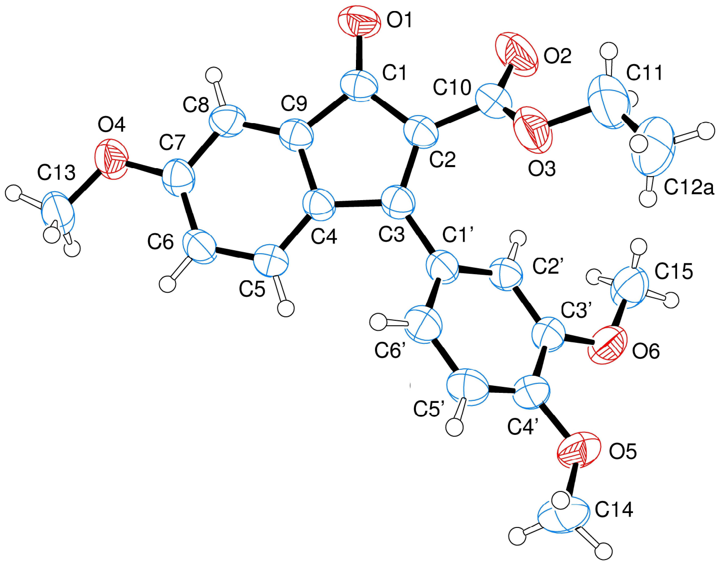

Figure S22. X-ray crystal structure of **2d**. Site occupation factor of C12a is 0.54(1). Ellipsoids enclose 50% probability.

Ethyl 6-methoxy-1-oxo-3-(3,4,5-trimethoxyphenyl)-1*H*-indene-2-carboxylate (2e).

Compound **2e** (0.32 g, yield 88%) was obtained starting from compound **6e** (0.35 g, 0.91 mmol) as a dark-red solid using petroleum ether-ethyl acetate (9:1 v/v) as the eluent. An analytical sample was obtained by recrystallization from dichloromethane by slow evaporation (mp 143-145 °C). ^{1}H-NMR (400 MHz, $CDCl_3$): 1.20 (t, J = 7.1, 3H), 3.85 (s, 3H), 3.87 (s, 6H), 3.92 (s, 3H), 4.21 (q, J = 7.1, 2H), 6.77 (s, 2H) 6.84 (dd, J = 8.1, 2.4, 1H), 7.17 (m, 2H). MS(ESI): m/z 421 (M+Na^{+}).

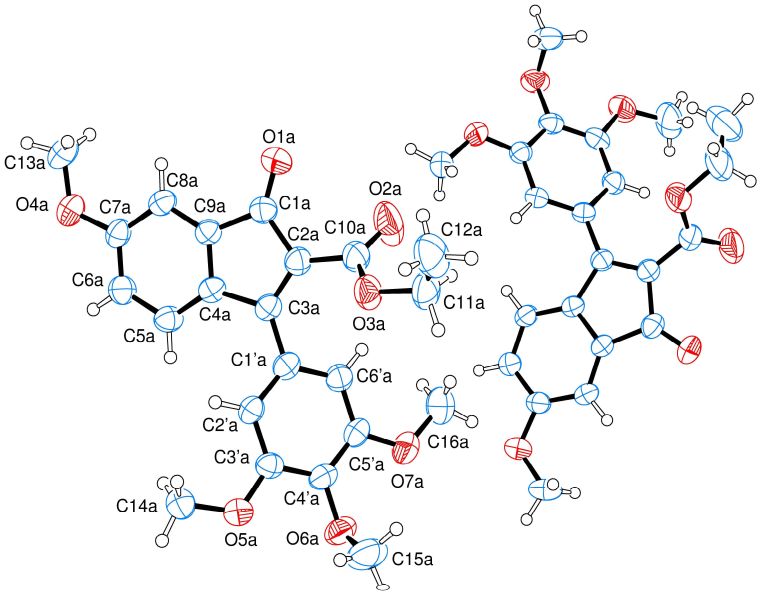

Figure S23. X-ray crystal structure of **2e**. Ellipsoids enclose 50% probability.

Ethyl 6-methoxy-3-(4-nitrophenyl)-1-oxo-1*H*-indene-2-carboxylate (2f).

Compound **2f** (0.39 g, yield 47%) was obtained starting from compound **6f** (0.79 g, 2.33 mmol) as a red solid using petroleum ether-ethyl acetate (8:2 v/v) as the eluent. An analytical sample was obtained by recrystallization from methanol by slow evaporation (mp 175-176 °C). ^{1}H-NMR (400 MHz, $CDCl_3$): 1.17 (t, J = 7.1, 3H), 3.87 (s, 3H), 4.18 (q, J = 7.1, 2H), 6.84 (dd, J = 8.2, 2.3, 1H), 6.93 (d, J = 8.2, 1H), 7.19 (d, J = 2.3, 1H), 7.67 (d, J = 8.6, 2H), 8.36 (d, J = 8.7, 2H). MS(ESI): m/z 376 ($M+Na^+$).

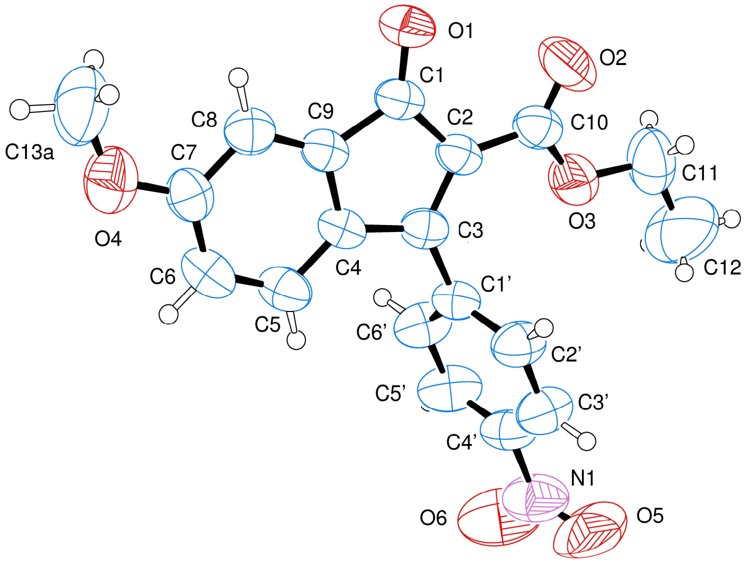

Figure S24. X-ray crystal structure of **2f**. Site occupation factor of C13a is 0.65(1). Ellipsoids enclose 50% probability.

Ethyl 3-(furan-3-yl)-6-methoxy-1-oxo-1*H*-indene-2-carboxylate (2h).

Compound **2h** (0.074 g, yield 89%) was obtained starting from compound **6h** (0.080 g, 0.28 mmol) as a red solid using petroleum ether-ethyl acetate (9:1 v/v) as the eluent. An analytical sample was obtained by recrystallization from diethyl ether by slow evaporation (mp 138-139 °C). ^{1}H-NMR (400 MHz, $CDCl_3$), 1.33 (t, J = 7.1, 3H), 3.86 (s, 3H), 4.31 (q, J = 7.1, 2H), 6.82 (s, 1H), 6.87 (dd, J = 8.2, 2.4, 1H), 7.16 (d, J = 2.3, 1H), 7.32 (d, J = 8.2, 1H), 7.58 (s, 1H), 8.18 (s, 1H). MS (ESI): m/z 321 ($M+Na^{+}$).

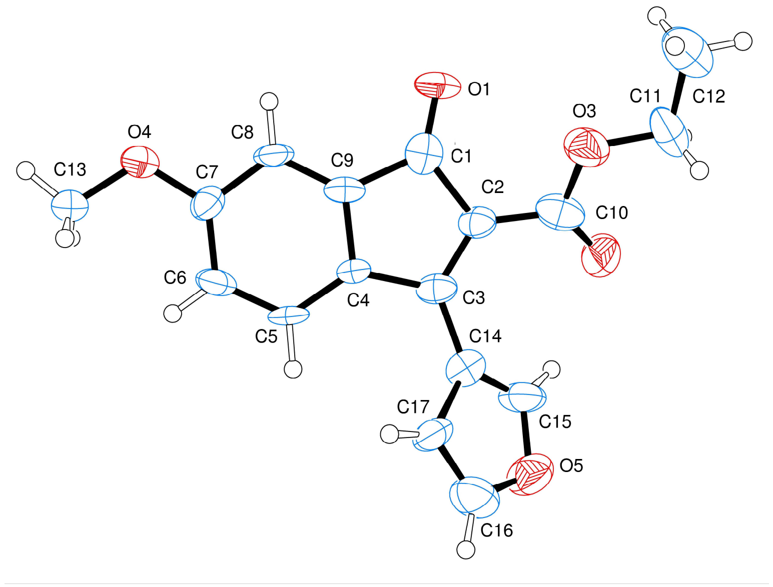

Figure S25. X-ray crystal structure of **2h**. Ellipsoids enclose 50% probability.

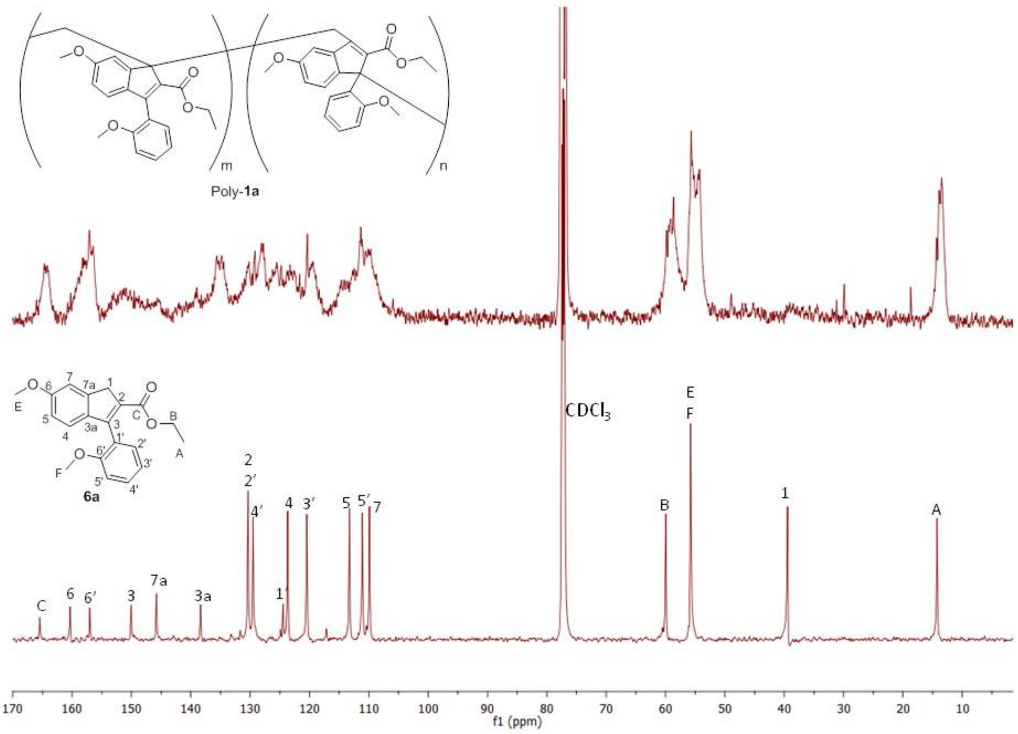

Figure S26. ^{13}C-NMR spectra in $CDCl_3$ of poly-**1a** and model compound **6a**.

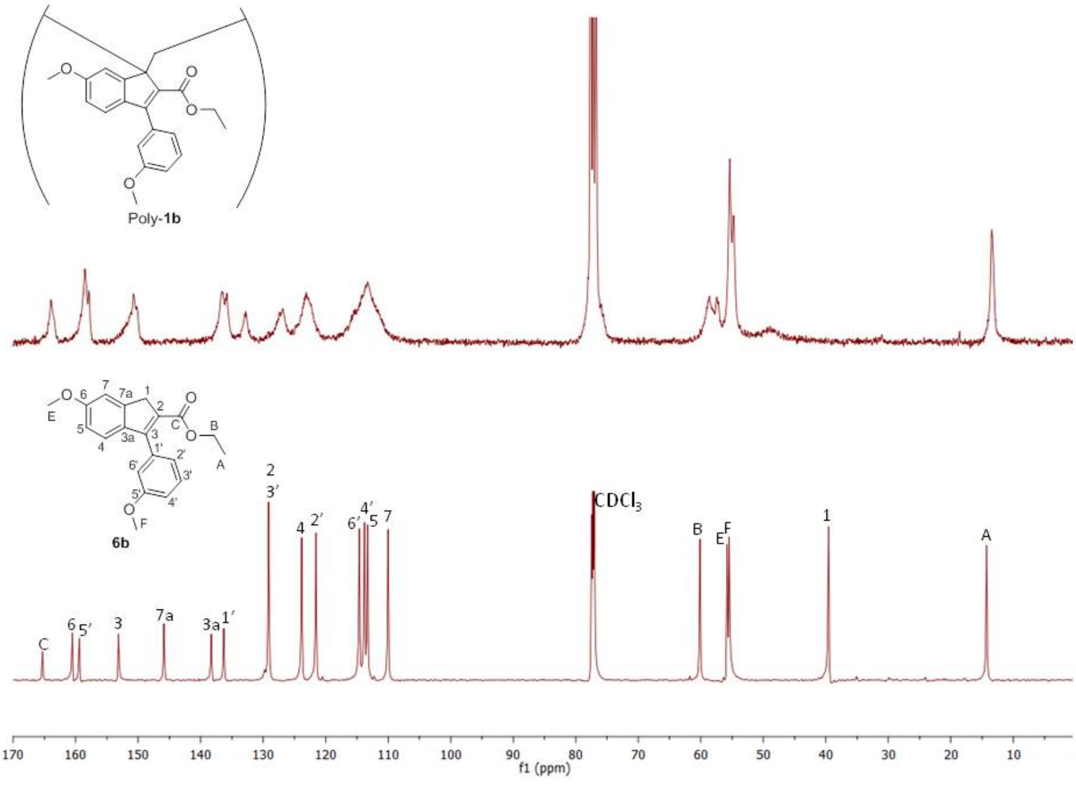

Figure S27. ^{13}C-NMR spectra in $CDCl_3$ of poly-**1b** and model compound **6b**.

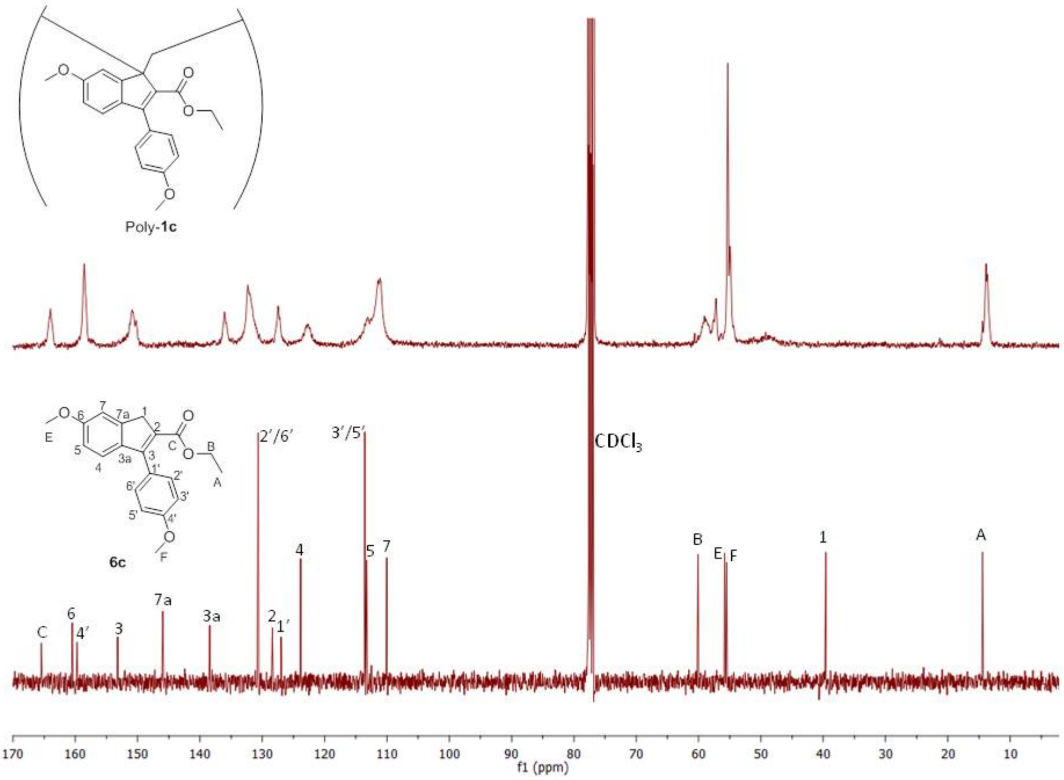

Figure S28. ^{13}C-NMR spectra in $CDCl_3$ of poly-**1c** and model compound **6c**.

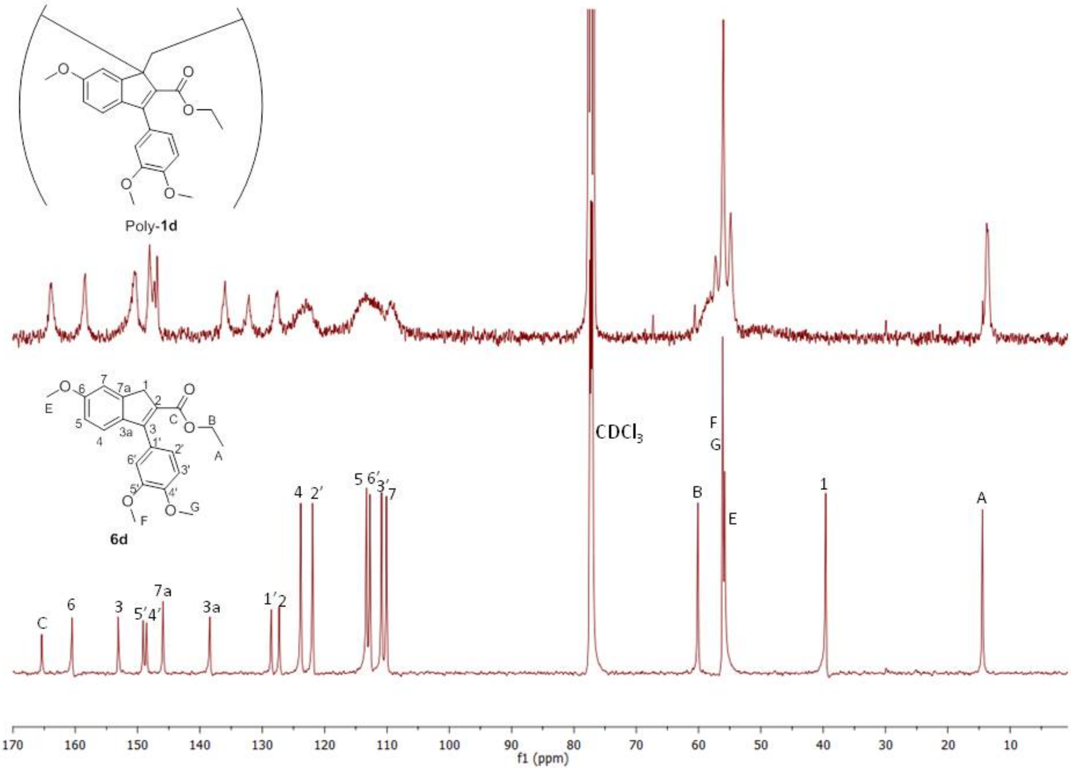

Figure S29. ^{13}C-NMR spectra in $CDCl_3$ of poly-**1d** and model compound **6d**.

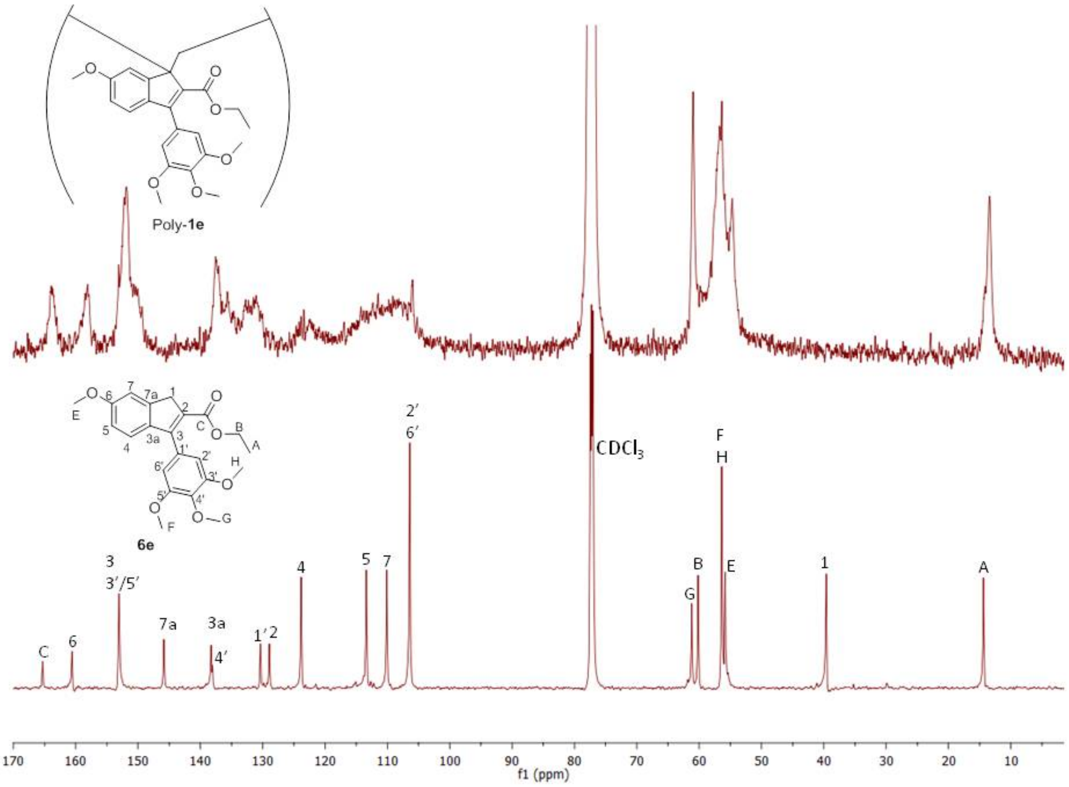

Figure S30. ^{13}C-NMR spectra in $CDCl_3$ of poly-**1e** and model compound **6e**.

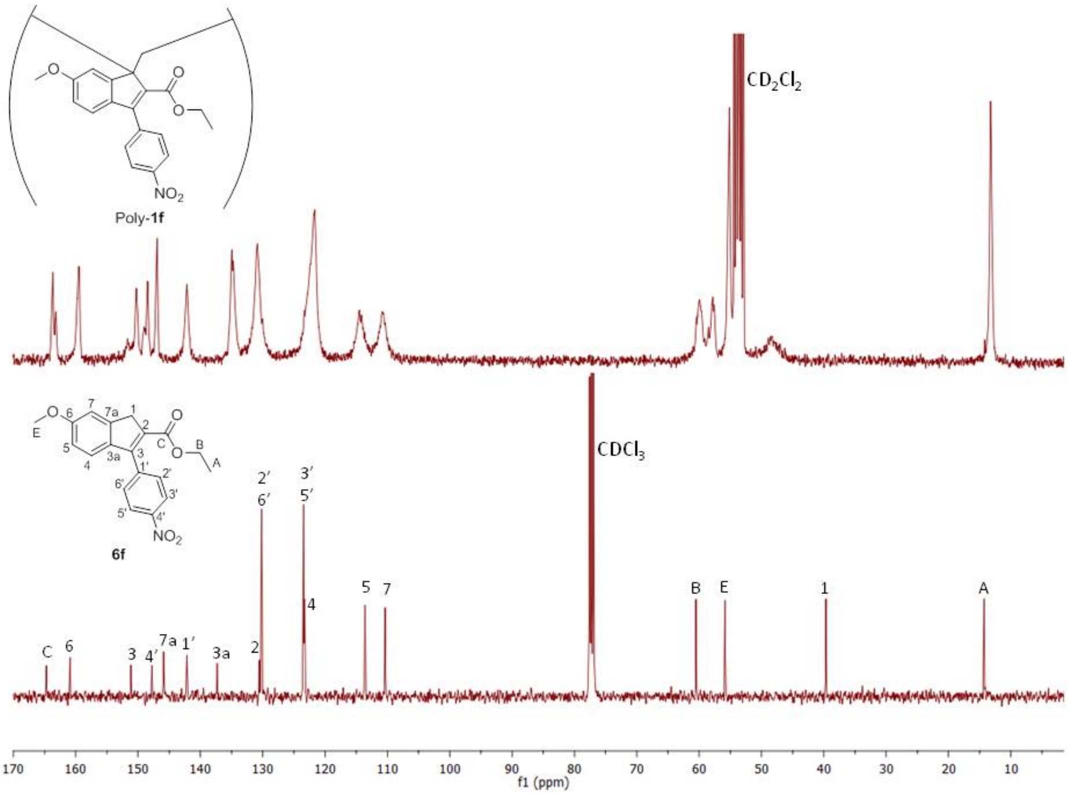

Figure S31. ^{13}C-NMR spectra of poly-**1f** (CD_2Cl_2) and model compound **6f** ($CDCl_3$).

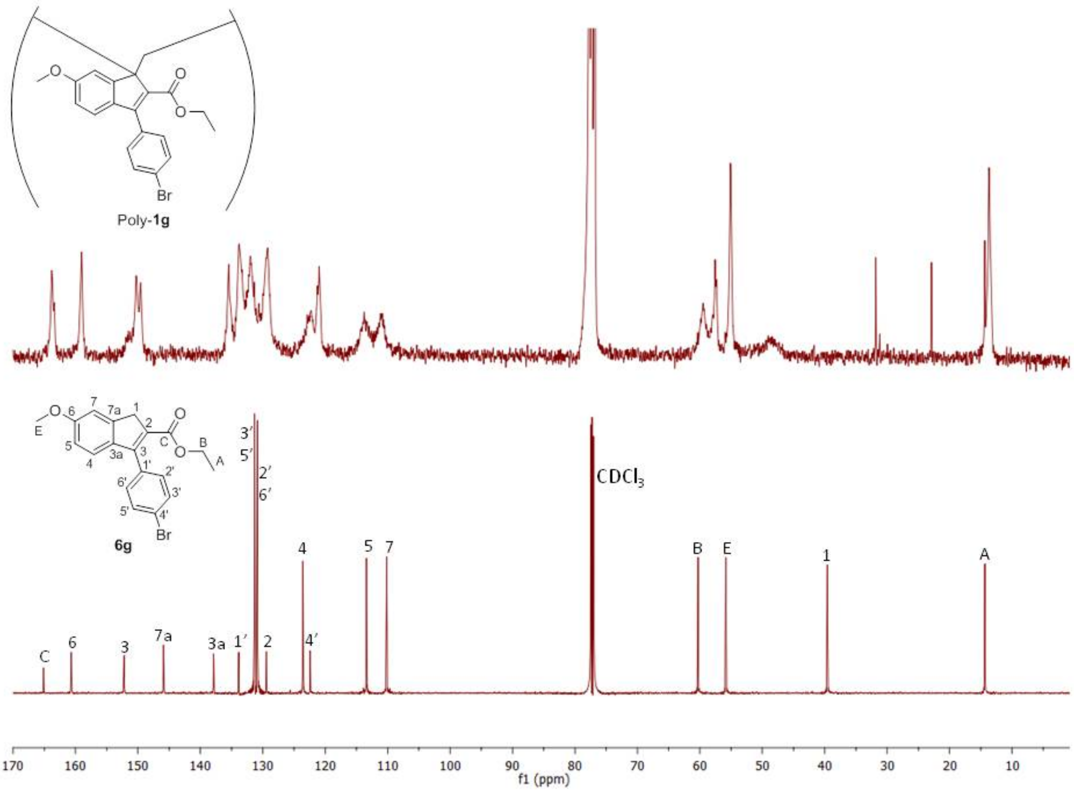

Figure S32. ^{13}C-NMR spectra in $CDCl_3$ of poly-**1g** and model compound **6g**.

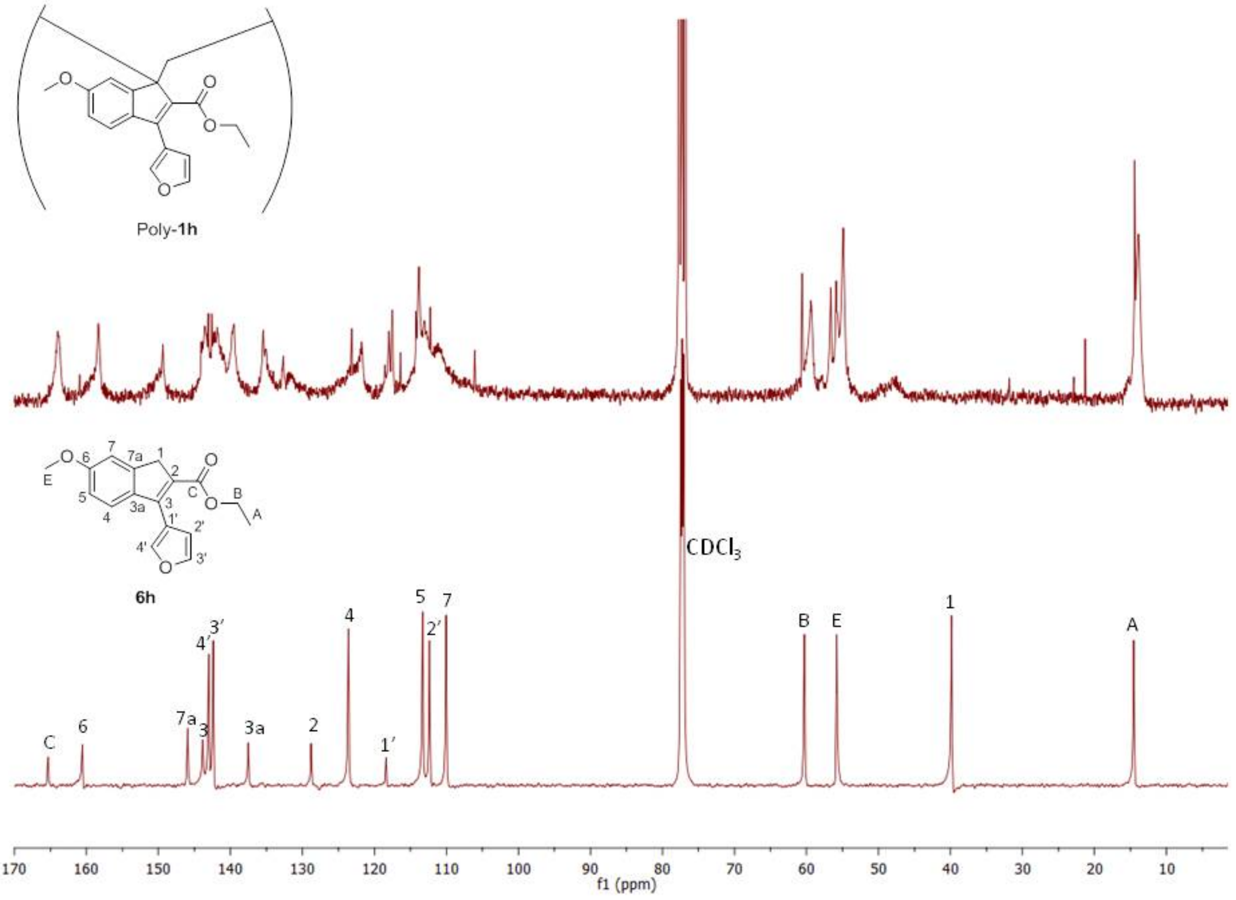

Figure S33. ^{13}C-NMR spectra in $CDCl_3$ of poly-**1h** and model compound **6h.**

References

1. Cappelli, A.; Villafiorita-Monteleone, F.; Grisci, G.; Paolino, M.; Razzano, V.; Fabio, G.; Giuliani, G.; Donati, A.; Mendichi, R.; Boccia, A. C.; Pasini, M.; Botta, C. Highly emissive supramolecular assemblies based on π-stacked polybenzofulvene hosts and a benzothiadiazole guest. *J. Mater. Chem. C* **2014**, *2*, 7897-7905.
2. Ahn, J. H.; Shin, M. S.; Jung, S. H.; Kang, S. K.; Kim, K. R.; Rhee, S. D.; Jung, W. H.; Yang, S. D.; Kim, S. J.; Woo, J. R.; Lee, J. H.; Cheon, H. G.; Kim, S. S. Indenone derivatives: a novel template for peroxisome proliferator-activated receptor γ (PPARγ) agonists. *J. Med. Chem.* **2006**, *49 (15)*, 4781–4784.
